# Configurations of Quality Dimensions Associated With Researcher and Patient Ratings of HIV/AIDS Mobile Health Apps in China: Fuzzy-Set Qualitative Comparative Analysis

**DOI:** 10.2196/88896

**Published:** 2026-10-22

**Authors:** Peng Liu, Fuzhi Wang, Changzheng Yang

**Affiliations:** 1 School of Medical Informatics and Engineering Bengbu Medical University Bengbu China; 2 School of Journalism and Communication Central China Normal University Wuhan, Hubei China

**Keywords:** HIV/AIDS, mobile health, mHealth, app quality, fsQCA, patient evaluation, MARS, uMARS, qualitative comparative analysis, fuzzy-set qualitative comparative analysis, Mobile App Rating Scale, user version of Mobile App Rating Scale

## Abstract

**Background:**

Mobile health (mHealth) apps have become important tools for HIV/AIDS management. Although the Mobile App Rating Scale and its user version are widely used, how different stakeholder groups combine quality dimensions into overall judgments remains unclear. Discrepancies between researcher and patient evaluations may affect app adoption and clinical effectiveness.

**Objective:**

This exploratory study aimed to compare configurational pathways associated with high subjective quality ratings of HIV/AIDS apps between researchers and patients using fuzzy-set qualitative comparative analysis.

**Methods:**

Twenty-one Chinese HIV/AIDS mobile apps from early 2023 were evaluated by 3 researchers using the original Mobile App Rating Scale and 2 HIV-positive patients using the user version of the Mobile App Rating Scale. Six conditions were examined: engagement, functionality, aesthetics, information quality, privacy protection, and developer type. Necessary and sufficient configurations were identified for both groups. Wilcoxon signed-rank tests compared raw ratings between groups. Sensitivity analyses covered 4 calibration anchor sets, 4 raw-consistency cutoffs, 4 proportional reduction in inconsistency cutoffs, and multivalue operationalization of privacy protection.

**Results:**

Researcher and patient ratings differed significantly for functionality (median difference 0.50, IQR 0.00 to 0.63; *P*=.003) and engagement (−0.20, IQR −0.40 to 0.10; *P*=.04), but not for aesthetics (*P*=.16), information (*P*=.07), or subjective quality (*P*=.06). Five sufficient configurations were identified for researchers (solution consistency=0.981, coverage=0.649) and 4 for patients (solution consistency=0.998, coverage=0.796). Configurations including privacy protection appeared only among researchers; its absence appeared in 3 patient configurations, reflecting the rarity of protection in the sample (5/21 apps) rather than lower patient ratings for protected apps. For patients, functionality (0.907) and information quality (0.915) exceeded the necessity threshold but were strongly correlated (*r*=0.816), so functionality was retained on substantive grounds rather than as an independent necessary condition. Illustrative cases were Wu Ai Fang Hua and Ai Yi Jian for the researcher-oriented pathway and Chabei for the patient-oriented pathway. Sensitivity analyses left the configurations unchanged across all anchor sets and raw-consistency cutoffs; 1 researcher configuration did not survive the stricter proportional reduction in inconsistency cutoffs, and the patient necessity result was anchor-dependent.

**Conclusions:**

The 2 evaluator panels’ assessments of HIV/AIDS mHealth apps appeared to follow different configurational logic in this sample, with several equifinal pathways to high subjective quality in each group. Because the patient panel comprised 2 evaluators, and 21 apps were analyzed with 6 conditions, the findings should be read as hypothesis-generating rather than representative of people living with HIV. The findings nonetheless suggest that both expert and end-user perspectives are needed during mHealth development, and that, because privacy protection was rare and not required for a favorable patient judgment, its provision in a population highly vulnerable to disclosure-related harm needs to be ensured by developers and regulators rather than left to user demand.

## Introduction

### Background

The global HIV/AIDS epidemic continues to pose significant public health challenges, with approximately 39 million people living with HIV worldwide as of 2022 [1]. Mobile health (mHealth) apps have emerged as innovative tools to support HIV prevention, treatment adherence, and self-management [2,3]. These digital interventions offer potential benefits including improved medication adherence, enhanced patient-provider communication, and increased access to health information [4,5]. Health app use is now widespread, but the behavioral functionality actually implemented in these apps varies widely [6,7].

The quality of mHealth apps is crucial for their effectiveness and user adoption [8,9]. Various stakeholders, including researchers, health care providers, and patients, evaluate app quality from different perspectives, potentially leading to divergent assessments [10]. Researchers typically use standardized evaluation frameworks emphasizing technical functionality, evidence-based content, and privacy protection [11,12]. In contrast, patients may prioritize usability, engagement features, and personal relevance [13,14].

The Mobile App Rating Scale (MARS) has become a widely adopted tool for assessing mHealth app quality across 4 dimensions: engagement, functionality, aesthetics, and information quality [15]. The user version of the Mobile App Rating Scale (uMARS) was subsequently developed to enable end users to evaluate apps from their own perspective [16,17]. While both versions provide comprehensive evaluation frameworks, they do not explicitly address how different evaluator groups weight these dimensions or how they combine to produce overall quality judgments. In HIV/AIDS care, this omission is consequential, because apps in this field must simultaneously deliver clinical utility and protect a health status that carries a high risk of stigma and discrimination [18,19].

### Theoretical Framework

#### Overview

Three complementary theoretical perspectives motivate the comparison undertaken here and were used to select conditions and to interpret results.

#### Task-Technology Fit and Technology Acceptance

Task-technology fit theory holds that a technology improves performance to the extent that its capabilities match the requirements of the user’s task [20]. The unified theory of acceptance and use of technology (UTAUT) and its consumer extension (UTAUT2) further specify performance expectancy, effort expectancy, and hedonic motivation as proximal determinants of use intention [21,22]. For a person living with HIV, the salient tasks are concrete: booking an anonymous test, obtaining reliable information, tracking medication. Performance expectancy and effort expectancy therefore map closely onto the MARS functionality and engagement dimensions and predict that these should dominate patient judgments. Researchers, who rate apps without a personal task to accomplish, are instead trained to apply normative criteria, so their judgments should weight information quality and safeguards more heavily.

#### Privacy Calculus and the Privacy Paradox

Privacy calculus theory models disclosure as a trade-off in which anticipated benefits are weighed against perceived risk [23]. The trade-off is systematically asymmetric when risks are abstract and delayed while benefits are immediate and concrete, producing the well-documented gap between stated privacy concern and observed behavior [24]. This framework predicts that privacy protection will function as a normative criterion for expert raters but will be discounted by end users, and it makes the direction of any observed divergence theoretically interpretable rather than merely descriptive.

#### eHealth Literacy and the Evaluator Effect

eHealth literacy encompasses the skills required to appraise and apply digital health information [25]. Features such as data-minimization practices and consent mechanisms are largely invisible in the interface and require appraisal skills that end users typically lack; visible features such as layout and interactivity do not. Independently of literacy, the usability literature documents an “evaluator effect”: trained evaluators applying the same instrument to the same artifact reach substantially different conclusions [26]. Together these perspectives imply that expert and user ratings should not be treated as interchangeable estimates of a single latent quality, which is the assumption implicitly embedded in current app-recommendation practice.

#### Configurational Logic

Each of the 3 perspectives outlined in the Theoretical Framework section describes a combination rather than an isolated effect: fit depends on functionality and content matching a task; the privacy calculus depends on risk relative to benefit. Conventional regression estimates the average, independent, symmetric contribution of each predictor [27,28] and cannot represent such conjunctions. Set-theoretic methods represent them directly through equifinality, conjunctural causation, and asymmetry [29-32].

### Gap in Knowledge

Despite growing recognition of the importance of patient perspectives in mHealth evaluation, few studies have systematically compared how researchers and patients evaluate app quality using configuration-based approaches [17]. Previous research has examined individual predictors of app quality ratings [33,34] but has not addressed: (1) whether multiple combinations of conditions are associated with high quality ratings (equifinality), (2) whether the same condition contributes differently depending on the conditions it is combined with (conjunctural causation), and (3) how evaluation patterns differ between professional researchers and end users. Studies in the HIV/AIDS app domain specifically have quantified mean quality scores and interrater agreement [35-39] but have not examined how dimensions combine. Furthermore, HIV/AIDS populations face disease-related stigma, acute privacy concerns about sensitive health information, and specific self-management needs that may shape their quality perceptions differently from general user populations [18,19,40].

### Study Objectives

This exploratory study aimed to: (1) identify the configurational pathways associated with high subjective quality ratings in HIV/AIDS apps as evaluated by researchers and by patients; (2) compare necessary and sufficient conditions between the two evaluator groups; (3) examine the degree of alignment or divergence in evaluation patterns; and (4) derive design-relevant propositions for HIV/AIDS app development that address both normative rigor and the user-side priorities observed in this panel. Given the panel and case numbers described in the Evaluator Panels and Sample Size Considerations section, the objective is to generate structured hypotheses about how quality judgments are formed, not to estimate population-level parameters.

## Methods

### Study Design

#### Overview

This cross-sectional study used a comparative evaluation design to assess HIV/AIDS mobile apps. Two distinct evaluator groups were recruited: (1) professional researchers with expertise in medical informatics, computer science, and social medicine; and (2) individuals living with HIV/AIDS. The study was conducted between February and May 2023, following PRISMA (Preferred Reporting Items for Systematic Reviews and Meta-Analyses) guidelines for systematic app selection [41]. The ratings analyzed here were collected for, and descriptively reported in, a previous quality evaluation of the same 21 apps [35]; the present study is a secondary, configurational analysis of that dataset, but the fuzzy-set qualitative comparative analysis (fsQCA), Wilcoxon, and sensitivity analyses reported here are new.

#### Unit of Analysis and Human Participation

Two distinct matters are addressed separately here. First, the unit of analysis is the mobile app: the 21 apps are the cases on which every datum is indexed, and all calibration, necessity, sufficiency, and coverage parameters refer to sets of apps rather than to sets of people. The evaluators are not cases; within this design each evaluator functions as a measurement instrument applied to every app, which is why evaluator reliability is assessed as interrater agreement rather than treated as sampling error. Second, and separately, 5 individuals produced those measurements, 2 of them people living with HIV. The first point concerns how the ratings are analyzed; the second concerns what was owed to the individuals who produced them. The first is set out in the Evaluator Panels and Sample Size Considerations section, and the second in the Recruitment and Eligibility, Data Collected, Basis on Which Prospective Ethics Review Was Not Sought, and Protections Applied sections.

#### Recruitment and Eligibility

The 2 patient evaluators were recruited entirely through the HIV prevention and control team of the local Center for Disease Control and Prevention (CDC), which is the institution that maintains the follow-up records for people living with HIV in this region. The research team specified the eligibility criteria; the CDC team applied them within its own records, identified individuals who met them, and invited participation. The research team did not receive the follow-up register, did not screen or select individuals, and did not at any stage collect, request, verify or record the serostatus of either evaluator, nor their names, contact details, age, sex, place of residence or any other personal information. Serostatus was therefore never an item of information held by the research team: it was a condition applied by the CDC within data the CDC already held for its own statutory purposes. Written informed consent was obtained from both evaluators before participation; in order to protect their privacy, the consent process was conducted by the CDC team, which collected and retained the signed consent forms on behalf of the study, so that no document bearing a participant’s name passed to the research team. What the research team received were completed rating forms, identified by a study code.

#### Data Collected

The data generated in this study were MARS and uMARS scores for apps that are publicly listed in Chinese app stores and in WeChat. No biological sample, medical record, clinical measurement, health-record extract, or laboratory result was obtained from any evaluator, and no demographic, behavioral, or health variable describing an evaluator entered the dataset or any analysis. The research team therefore collected no personal information of any kind about the evaluators; the only data collected were ratings of publicly available software, held under study codes on an encrypted institutional server.

#### Basis on Which Prospective Ethics Review Was Not Sought

Prospective ethics review was not applied for, and the basis for that is stated here so that readers can assess it. The applicable instrument is the Measures for Ethical Review of Life Science and Medical Research Involving Humans, issued jointly by the National Health Commission, the Ministry of Education, the Ministry of Science and Technology, and the National Administration of Traditional Chinese Medicine (Guo Wei Ke Jiao Fa [2023] No. 4) [42] and in force from 18 February 2023, that is, for the whole of the evaluation period. Article 3 defines the scope of the Measures as research conducted with human beings as subjects, or conducted using human biological samples or personal information data, including health records and behavioral data; Article 50 defines a research participant correspondingly as a person who provides biological samples, information data, health records or behavioral data for the research. On both definitions, this study lies outside the scope of the Measures: no human being was a subject of investigation, no biological sample was used, and no personal information data, health record, or behavioral record of any evaluator was provided to, held by, or analyzed by the research team. What the evaluators supplied were ratings of external artifacts, which are properties of the apps rather than information about the raters. Alternatively, were the ratings to be regarded as information data supplied by identifiable individuals, Article 32(2) provides that research using anonymized information data may be exempt from ethics review where it causes no bodily harm and involves neither sensitive personal information nor commercial interest; each condition is satisfied here, since ratings were coded at source, no procedure was applied to any person, no sensitive personal information reached the research team, and the study had neither commercial sponsorship nor commercial interest. The risk to participants was no greater than the minimal-risk threshold at which Article 31 permits review by expedited procedure.

This reporting position is the prevailing one in the MARS/uMARS app-quality literature. App-quality evaluations of this design are routinely published without prospective ethics review on the ground that the object of evaluation is publicly available software rather than persons [43], and the same holds where lay end users rather than experts complete the instrument: in the Greek validation of the uMARS, in which 91 informed participants each rated an app twice, the authors reported that ethics approval was not required because the study consisted of completion of the uMARS and involved no clinical procedure [44].

#### Protections Applied

Although the study lies outside the scope of mandatory review, the protections owed to participants were applied in full and in accordance with the principles of the Declaration of Helsinki. Both patient evaluators were informed of the purpose, procedures, and time commitment of the study and of their right to withdraw at any point without giving a reason and without consequence for their care, and both gave written informed consent before participating; the signed forms were collected and retained by the CDC team on their behalf. Recruitment through the CDC follow-up channel was chosen because it is the only route by which people living with HIV in this region can be approached without their status being disclosed to a third party. No information capable of identifying an evaluator appears in this article or in any associated data file.

### App Selection and Data Collection

A systematic search was conducted on the Qimai app data platform, Aladdin WeChat applet data platform, and WeChat from February 18-19, 2023. Search terms included “HIV”, “AIDS”, “Human Immunodeficiency Virus”, “Acquired Immunodeficiency Syndrome”, and their Chinese equivalents. The Qimai Keyword Expansion Assistant was used to identify relevant apps with a relevance score exceeding 50% and a search index surpassing 4605.

WeChat applets, lightweight apps embedded within the WeChat platform, were included due to their widespread use in China. The Aladdin Index platform was used to identify relevant WeChat applets. Additionally, a snowball sampling method was implemented through collaboration with local CDC HIV prevention teams to identify apps commonly used within the HIV/AIDS community but potentially missed by platform searches.

The exclusion criteria for this study were the following: (1) non-Simplified Chinese language, (2) games, (3) apps with limited functionality, (4) apps not primarily focused on HIV/AIDS content, (5) more than 50% inaccessible content, (6) terminated services, (7) apps restricted to internal use, and (8) apps primarily intended for advertising or product sales. Following systematic screening, 21 HIV/AIDS apps were included for evaluation. The selection process and the subsequent 2-panel evaluation design are shown in Figure 1 [35].

**Figure 1:**
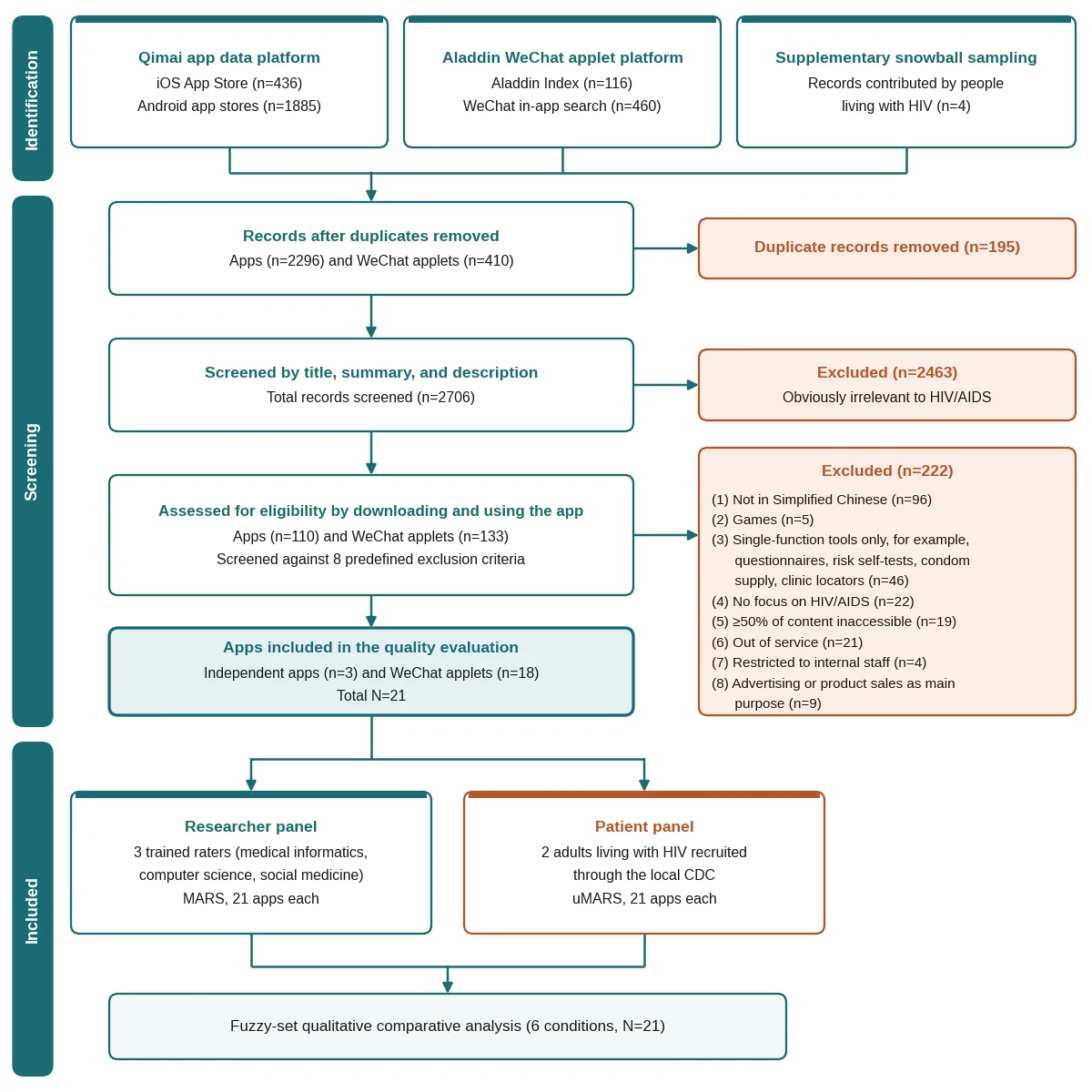
Flow diagram of the identification, screening, and inclusion of Chinese HIV/AIDS mobile apps, and of the 2-panel evaluation design [35]. CDC: Center for Disease Control and Prevention; MARS: Mobile App Rating Scale; uMARS: user version of Mobile App Rating Scale.

### Evaluation Tools: MARS and uMARS

Two versions of the MARS were used in this study to accommodate the different evaluator groups. The original MARS was used for researcher evaluations, while uMARS was used for patient evaluations [15,17].

The original MARS is a validated 23-item scale designed for expert assessment of app quality across four objective dimensions: (1) engagement (entertainment, interest, customization, interactivity, and target group); (2) functionality (performance, ease of use, navigation, and gestural design); (3) aesthetics (layout, graphics, and visual appeal); and (4) information (quality, quantity, credibility, and evidence-base). Each item is rated on a 5-point scale (1=inadequate to 5=excellent). MARS also includes a subjective quality subscale, which served as the outcome variable in this study [15].

The uMARS is a 20-item adaptation designed for end user evaluation, retaining the same 4 dimensions but with simplified language and reduced technical terminology to enhance accessibility for nonexpert raters [17]. Both MARS and uMARS have demonstrated good reliability and validity across diverse mHealth apps and languages [45-47]. The use of parallel instruments allowed for meaningful comparison between expert and user perspectives while ensuring each group could evaluate apps using an appropriate tool.

### Evaluator Panels and Sample Size Considerations

#### Researcher Panel

Three researchers conducted the app evaluations using the original MARS and were purposively selected to cover the 3 competence domains that MARS scoring requires: health information content, software engineering, and app-quality methodology. Rater 1 holds a master’s degree in Medical Informatics with extensive experience in medical information analysis. Rater 2 holds a master’s degree in Computer Science with software development expertise. Rater 3 holds a PhD in Social Medicine with substantial experience in mobile app evaluation.

Before formal evaluation, all 3 raters completed MARS training by reviewing instructional videos and conducting pilot evaluations on 4 randomly selected apps (2 WeChat applets and 2 independent apps). For the formal evaluation, raters 1 and 2 independently assessed all 21 apps. To ensure consistency, evaluators completed assessment of all apps for dimension N before proceeding to dimension N+1. When discrepancies occurred between raters 1 and 2, rater 3 reassessed the app, and a consensus score was reached through structured discussion.

#### Patient Panel

A total of 2 patient evaluators were recruited through collaboration with local CDC HIV prevention and control teams. Eligibility criteria, applied by the CDC team within its own follow-up records rather than by the research team, were the following: (1) confirmed HIV-positive diagnosis on the CDC follow-up register; (2) age ≥18 years; (3) ability to read and understand Simplified Chinese; and (4) experience using mobile apps. Both participants provided written informed consent, which the CDC team collected and retained on behalf of the study to protect their privacy, and both were assured of anonymity and data confidentiality; no serostatus information and no personal identifiers were transferred to the research team at any stage (see the Study Design and Ethical Considerations sections). Recruitment was purposive rather than probabilistic, with the aim of obtaining evaluators whose lived experience made them able to judge the apps as intended users.

Recruiting patient evaluators proved substantially more difficult and time-consuming than recruiting expert raters, and the size of the patient panel reflects that difficulty rather than a design preference. People living with HIV are a small and highly stigmatized minority in China, and they attach exceptional importance to the confidentiality of their serostatus. The dominant concern voiced by those who were approached and declined was not the time required by the rating protocol but the possibility that participation would create a record linking them by name to HIV-related research, however strong the assurances of anonymity. Recruitment could proceed only through the local CDC’s routine follow-up visits, which is the sole channel through which people living with HIV in this region can be approached without their status being disclosed to a third party, and a considerable period elapsed before 2 individuals willing to take part were identified. No demographic detail about the 2 participants is reported, because none was collected: in a 2-person panel recruited through a single follow-up program, age, sex and city in combination would be potentially identifying, and the research team therefore asked the CDC team not to transfer such details.

Patient evaluators completed the uMARS rather than the original MARS. They were provided with brief uMARS training materials adapted for nontechnical users, emphasizing the evaluation of user experience rather than technical specifications. Patients independently evaluated all 21 apps over a 4-week period and submitted their ratings through a secure online platform. Adequate time was provided for patients to thoroughly explore each app before rating. Patient scores were averaged across the 2 evaluators for each app and dimension.

#### Sample Size Considerations

Two aspects of sample size warrant explicit justification: the number of evaluators and the number of apps.

#### Number of Evaluators

The use of 2 to 3 trained raters is not a constraint specific to this study but the established design convention of the MARS/uMARS app-quality literature, including in this journal. The MARS was developed and validated on independent ratings by a small expert panel and reported excellent interrater reliability under those conditions (intraclass correlation coefficient [ICC]=0.79) [15]. The convention has been maintained consistently since. Table 1 summarizes evaluator panel size across published MARS-based app-quality evaluations in *JMIR mHealth and uHealth* and closely related outlets, spanning instrument-validation studies, single-disease app reviews, and cross-national comparisons.

**Table 1:** Evaluator panel size in published MARS^a^-based app-quality evaluations. Studies are ordered by the number of apps evaluated.

Study	Health domain	Instrument	Apps evaluated (n)	Evaluators (n)
Miró and Llorens-Vernet [48], 2021	Chronic conditions (interrater reliability study)	MARS, MAG^b^	4	8
Fernández-Sánchez et al [36], 2025	HIV and STI^c^ prevention	MARS	6	2 (plus a third for disputes)
Shen et al [49], 2021	Drug-drug interaction checking	MARS	7	2
Schmeelk et al [50], 2022	COVID-19 symptom monitoring	MARS	20	2 (5 for calibration)
This study	HIV/AIDS	MARS, uMARS^d^	21	3 researchers; 2 patients
Mani et al [51], 2015	Mindfulness	MARS	23	2 (second rater on 30% subset)
Amor-García et al [52], 2020	Genitourinary cancer	MARS	46	2
Yamamoto et al [53], 2022	Mental health (instrument validation)	MARS-J^e^	50	2
Messner et al [47], 2020	Mental health (instrument validation)	MARS-G^f^	104	2 per app

^a^MARS: Mobile App Rating Scale.

^b^MAG: Mobile App Development and Assessment Guide.

^c^STI: sexually transmitted infection.

^d^uMARS: user version of the Mobile App Rating Scale.

^e^MARS-J: Japanese version of the Mobile App Rating Scale.

^f^MARS-G: German version of the Mobile App Rating Scale.

Two features of this evidence are relevant here. First, panel size does not scale with the number of apps: 2 raters were used whether 6, 7, 20, 23, 46, 50, or 104 apps were evaluated [36,47,49-53]. This is because the rater in this paradigm functions as a measurement instrument applied to each app rather than as a sampled unit of analysis. The unit of analysis is the app; the psychometric requirement is demonstrable agreement between raters, not a large rater sample. Reliability is accordingly evaluated as interrater agreement, as reported in the Interrater Agreement section, rather than treated as sampling error. Second, 3 raters—the number used for our researcher panel—is at the upper rather than the lower end of the distribution [35,54]; most comparable studies use two. Beyond the studies tabulated, the same convention is evident across app domains in this journal, including drug-drug interaction checkers [55], medication adherence apps [56], apps for deaf and hard-of-hearing users [57], hematological condition apps [58,59], traditional vs modern medicine apps in China [60], noncommunicable disease management apps [61], and apps for men who have sex with men in China [39], as well as in the Korean [46] and Arabic [62] MARS validations, in reviews of depression apps in German app stores [34] and of commercial weight-management apps [63], and in the 2 published MARS-based evaluations of HIV/AIDS apps [37,38].

The patient panel requires a separate justification, because the logic that supports a small expert panel does not transfer directly to end users: experts are trained to converge on a normative standard, whereas end users are informative partly because they differ from one another. Three considerations support the present design. First, HIV-positive status is a stigmatized and legally protected attribute in China, and recruitment is correspondingly difficult: people living with HIV could be approached only through CDC follow-up visits, and the prevailing reason for declining was concern that participation would create documentation linking the individual to HIV-related research. Second, both patient evaluators rated all 21 apps on all dimensions, so the design is complete rather than sparse: there are no missing cells, and each app is evaluated by the same evaluators, which is the condition under which paired comparison with the researcher panel is valid. Third, agreement between the 2 patient raters was examined empirically rather than assumed, and the limits of agreement were narrow relative to the rating range (see Statistical Analysis). We therefore treat the patient results as characterizing the configurational structure observed in this panel and frame them as exploratory and hypothesis-generating throughout.

#### Terminology

Because a 2-person panel cannot stand for a population, a fixed vocabulary is used throughout. The patient panel and the 2 patient evaluators refer to the 2 individuals who completed the uMARS; patient configurations and patient-side pathways refer to configurations recovered from those 2 evaluators’ averaged ratings of the 21 apps; and statements about the researcher panel are likewise restricted to the 3 raters described in the Researcher Panel section. No expression in this paper is intended to characterize the preferences of people living with HIV in China as a population, or of Chinese app users generally. Where the text refers to patient evaluations, it refers to this panel.

#### Number of Apps

Twenty-one apps are the complete set of Chinese HIV/AIDS apps meeting the inclusion criteria at the time of the search, identified from 2901 screened records. This is not a sample drawn from a larger population, but a census of an intentionally narrow population, and the small size is itself a substantive finding about the Chinese HIV/AIDS app market [35]. Comparable app-quality evaluations in this journal have analyzed as few as 4, 6, and 7 apps (Table 1), and evaluations of 20 apps have supported published quality conclusions [50].

For the configurational analysis specifically, qualitative comparative analysis (QCA) was developed for small- and intermediate-N designs and is conventionally applied to 10-50 cases, where intimate case knowledge is available, and each case can be interpreted individually [64,65]. With N=21, the present study lies within this range and, unlike most regression-based designs, permits every case to be inspected individually against its configuration. Six conditions with 21 cases nevertheless produce substantially limited diversity: of the 64 logically possible configurations, only 14 (researcher data) and 15 (patient data) contained at least one case with membership above 0.5. We therefore report the intermediate solution, which uses only easy counterfactuals [66,67]; interpret configurations alongside the specific apps that instantiate them; and report the sensitivity analyses described in the Robustness and Sensitivity Analyses section.

### Privacy Protection Assessment

Privacy protection was coded by 2 researchers independently from the apps themselves and their store listings. An app was coded as protected (code 1) only if it met all three of the following criteria: (1) a privacy policy accessible from within the app; (2) explicit disclosure of what data are collected and how they are used; and (3) user control over personal data, such as account or data deletion. All other apps were coded as unprotected (code 0). Coding disagreement occurred for 2 of 21 apps and was resolved by discussion with reference to the app interface; agreement before discussion was 90.5% (19/21).

We acknowledge that this binary treatment simplifies a multidimensional construct. Comprehensive instruments for privacy assessment in mHealth evaluate policy fairness against data-protection regulation, permission and data-sharing behavior, and technical safeguards, and they yield graded rather than dichotomous scores [68-71]. Two considerations motivated the binary coding here. First, the empirical distribution was strongly bimodal: apps either provided all 3 elements or none of them, and no app in this sample provided partial protection in the sense of a policy without disclosure or without user control. Second, a graded index estimated from 21 cases would add analytic flexibility without adding usable variance. To test whether this choice drove the results, we repeated the entire analysis with privacy specified as a 3-level fuzzy set (0=no protection; 0.5=policy, disclosure, and user control; 1=the preceding elements plus an in-app privacy password), reported in the Alternative Privacy Operationalization section.

### fsQCA

#### Conditions and Outcome

Six conditions were examined: engagement, functionality, aesthetics, and information quality (continuous, from MARS/uMARS); privacy protection (binary); and developer type (binary: 1=government organization, 0=nongovernment). The outcome was the subjective quality rating. Conditions were selected from the MARS/uMARS framework and from prior literature on determinants of mHealth app quality [11,15,33].

#### Calibration

Calibration transforms raw scores into fuzzy-set membership between 0 and 1 [66,72]. Continuous conditions were calibrated using the direct method with 3 anchors set at the 95th percentile (full membership), the 50th percentile (crossover), and the 5th percentile (full nonmembership). Percentile-based anchoring is standard where no external substantive benchmark exists for the measure, and is recommended when scores are instrument-specific and their absolute values are not interpretable against a criterion [73,74]. This applies here: a MARS engagement score of 3.0 has no external meaning as a threshold of “high engagement,” whereas its position within the observed distribution of HIV/AIDS apps does. Anchors were derived separately for the researcher and patient datasets, since the 2 instruments produce different rating distributions and a common anchor would confound instrument scaling with evaluator judgment. Membership scores falling exactly at 0.50 after calibration were adjusted upward by 0.001, so that no case was left at the point of maximum ambiguity; this mattered most for patient functionality, since 14 of the 21 apps received the same functionality rating and that rating is the crossover value. Where a percentile anchor coincided with the crossover, which occurred only for patient functionality under the wider anchor sets used in the sensitivity analysis, the affected anchor was set to the nearest observed value beyond the crossover. Binary conditions were retained as crisp sets. Because the choice of percentile anchors is a researcher decision rather than a property of the data, its consequences were examined explicitly in the sensitivity analysis.

#### Necessity

A condition is necessary if the outcome cannot occur without it [66,75]. Necessity was assessed by consistency, with the conventional threshold of 0.90 [67], and evaluated jointly with coverage to guard against trivial necessity. Both the presence and the absence of each condition were tested.

#### Sufficiency

Truth tables were constructed for both datasets. A frequency cutoff of 1 was applied, as is standard for small-N designs [64], together with a raw consistency cutoff of 0.80 and a proportional reduction in inconsistency (PRI) consistency cutoff of 0.70. Boolean minimization produced complex, parsimonious, and intermediate solutions; the intermediate solution is reported as the primary result [67]. Following Fiss [76], conditions appearing in both the parsimonious and the intermediate solution are designated core, and those appearing only in the intermediate solution are designated peripheral.

#### Robustness and Sensitivity Analyses

Four sets of checks were performed, following current QCA reporting recommendations [64,74,77]. First, all analyses were repeated under 4 calibration anchor sets (95/50/5, 90/50/10, 85/50/15, and 80/50/20 percentiles). Second, the truth table analysis was repeated across raw consistency cutoffs of 0.75, 0.80, 0.85, and 0.90 crossed with PRI cutoffs of 0.65, 0.70, 0.75, and 0.80. Third, the analysis was repeated with the 3-level privacy set described in the Privacy Protection Assessment section. Fourth, necessity consistencies were recomputed under each anchor set. The results are considered robust where the set of configurations and their substantive interpretation are unchanged, and consistency remains above the 0.80 threshold; any specification under which this does not hold is reported explicitly.

### Statistical Analysis

#### Overview

Descriptive and comparison statistics were computed in Python 3.12; calibration, necessity analysis, and truth-table analysis were conducted in fsQCA (version 4.1; University of California). Descriptive statistics were computed for all variables. Because the 2 evaluator groups rated the same 21 apps, differences in raw dimension scores were tested with Wilcoxon signed-rank tests, with the matched rank-biserial correlation as the effect size; the nonparametric test was chosen because dimension scores were bounded, skewed, and drawn from a small sample. Agreement between groups was quantified with Pearson and Spearman correlations. Given 5 prespecified comparisons, *P* values should be interpreted descriptively rather than as confirmatory tests.

#### Interrater Agreement

Agreement was examined within each panel separately, and because the 2 panels completed different instruments, no estimate reported in this section combines researcher and patient ratings. Within the researcher panel the 2 independent raters were raters 1 and 2, each of whom scored all 21 apps on the original MARS before any discrepancy was referred to rater 3 and before consensus scores were formed; Bland-Altman analysis of that MARS dataset [35,78] gave a mean difference of 0.01 (SD 0.10; 95% limits of agreement −0.19 to 0.21) for app quality and 0.07 (SD 0.49; 95% limits of agreement −0.89 to 1.03) for subjective quality. Within the patient panel, the 2 independent raters were the 2 patient evaluators whose ratings are analyzed in the present paper, each of whom scored all 21 apps on the uMARS before the 2 sets of scores were averaged; Bland-Altman analysis of that uMARS dataset gave a mean difference of 0.13 (SD 0.26; 95% limits of agreement −0.38 to 0.64) for app quality and 0.23 (SD 0.45; 95% limits of agreement −0.66 to 1.12) for subjective quality. Both uMARS estimates therefore derive specifically from the 2 patient evaluators included here, and no researcher rating enters them; conversely, the 2 MARS estimates derive only from raters 1 and 2, and no patient rating enters them.

Limits of agreement for the 2 app-quality scores are narrow relative to the 1-5 rating range, indicating close agreement within both panels; the wider limits for the single-item subjective quality scores are consistent with the greater variability expected of subjective judgments. Bland-Altman analysis was the prespecified agreement method in the parent evaluation, and it is the appropriate choice for 2 raters because it quantifies both systematic bias and the range of disagreement on the original measurement scale rather than expressing agreement relative to between-app variance [78]. An ICC could not be computed retrospectively for either panel, because the archived dataset retains only the postconsensus researcher scores and the averaged patient scores; the rater-level score files are no longer available, and the Bland-Altman estimates reported in this section are those computed in the parent evaluation while those files were still held [35]. Future apps of this design should archive rater-level scores so that both agreement statistics can be reported.

### Ethical Considerations

This study evaluated publicly available mobile apps. The data analyzed were MARS and uMARS ratings of those apps; no biological sample, clinical record, health record, or personal information of any evaluator was collected, held, or analyzed by the research team. The serostatus of the 2 patient evaluators was neither collected nor verified by the research team: eligibility was applied by the collaborating CDC HIV prevention and control team within its own follow-up records, and no identifier or personal detail was transferred to the research team. Prospective ethics review was not sought, on the basis set out in the Methods: the study lies outside the scope defined in Article 3 of the Measures for Ethical Review of Life Science and Medical Research Involving Humans (Guo Wei Ke Jiao Fa [2023] No. 4) [42] and would in the alternative fall within the exemption in Article 32(2) of the same instrument. This position is consistent with comparable app-quality evaluations, including those in which lay end users completed the instrument [43,44]. The study was nonetheless conducted in accordance with the principles of the Declaration of Helsinki: both patient evaluators were informed of the purpose and procedures of the study and of their right to withdraw at any time without consequence, and both provided written informed consent, the signed forms having been collected and retained by the CDC team on behalf of the study to protect the participants’ privacy.

## Results

### Descriptive Statistics and Group Comparison

Twenty-one HIV/AIDS apps were evaluated by both researcher and patient groups. Table 2 presents the descriptive statistics for all variables. Researcher ratings demonstrated moderate variability across conditions, with functionality receiving the highest mean score (4.28, SD 0.39) and engagement the lowest (mean 2.40, SD 0.53). Subjective quality ratings from researchers averaged 2.47 (SD 0.65), ranging from 1.13 to 4.13.

**Table 2:** Descriptive statistics for researcher (Mobile App Rating Scale) and patient (user version of the Mobile App Rating Scale) ratings of 21 HIV/AIDS apps [35].

Variable	R^a^ mean (SD)	R min^b^	R max^c^	P^d^ mean (SD)	P min	P max
Engagement	2.40 (0.53)	1.60	3.60	2.56 (0.63)	1.80	4.70
Functionality	4.28 (0.39)	3.25	4.75	3.84 (0.59)	1.50	4.50
Aesthetics	3.40 (0.49)	2.33	4.17	3.22 (0.48)	1.67	4.17
Information	3.83 (0.33)	3.33	4.33	3.90 (0.74)	1.75	4.75
Subjective quality	2.47 (0.65)	1.13	4.13	2.75 (0.61)	1.00	3.75

^a^R: researcher.

^b^min: minimum.

^c^max: maximum.

^d^P: patient.

Patient evaluations showed similar patterns but with notable differences. Patients rated engagement slightly higher than researchers (mean 2.56, SD 0.63), though still the lowest among all dimensions. Information quality received the highest patient rating (mean 3.90, SD 0.74), followed by functionality (mean 3.84, SD 0.59). Patient subjective quality ratings averaged 2.75 (SD 0.61), ranging from 1.00 to 3.75, indicating somewhat higher overall satisfaction compared to researchers.

Wilcoxon signed-rank tests on the paired ratings (Table 3) showed that the 2 groups differed significantly on functionality, which researchers rated substantially higher than patients (median difference 0.50, IQR 0.00 to 0.63; *W*=14.0, *P*=.003, rank-biserial *r*=0.82), and on engagement, which patients rated slightly higher (median difference −0.20, IQR −0.40 to 0.10; *W*=57.5, *P*=.04, *r*=−0.50). Differences in information quality (*P*=.07), subjective quality (*P*=.06), and aesthetics (*P*=.16) did not reach significance. Rank correlations between the groups were moderate for engagement (ρ=0.64, *P*=.002) and information (ρ=0.63, *P*=.002) but near 0 for functionality (ρ=0.20, *P*=.38). Functionality is thus the dimension on which the groups both differ most in level and agree least in ordering, whereas engagement is rated similarly and ranked consistently. This pattern provides the empirical warrant for analyzing the 2 groups separately rather than pooling them. Dimension-level differences between the 2 groups are displayed in Figure 2.

**Table 3:** Comparison of researcher and patient ratings across the same 21 apps (Wilcoxon signed-rank tests and rank correlations).

Dimension	R^a^ mean (SD)	P^b^ mean (SD)	Median difference (IQR)	*W* ^c^	*P* value	Rank-biserial *r*	Pearson *r*	Spearman ρ
Engagement	2.40 (0.53)	2.56 (0.63)	−0.20 (−0.40 to 0.10)	57.5	.04	−0.50	0.71	0.64
Functionality	4.28 (0.39)	3.84 (0.59)	0.50 (0.00 to 0.63)	14.0	.003	0.82	0.06	0.20
Aesthetics	3.40 (0.49)	3.22 (0.48)	0.17 (−0.17 to 0.51)	67.5	.16	0.36	0.23	0.32
Information	3.83 (0.33)	3.90 (0.74)	−0.21 (−0.42 to 0.01)	56.0	.07	−0.47	0.48	0.63
Subjective quality	2.47 (0.65)	2.75 (0.61)	−0.38 (−0.75 to 0.25)	54.0	.06	−0.49	0.39	0.45

^a^R: researcher.

^b^P: patient.

^c^*W*: Wilcoxon signed-rank test statistic.

**Figure 2:**
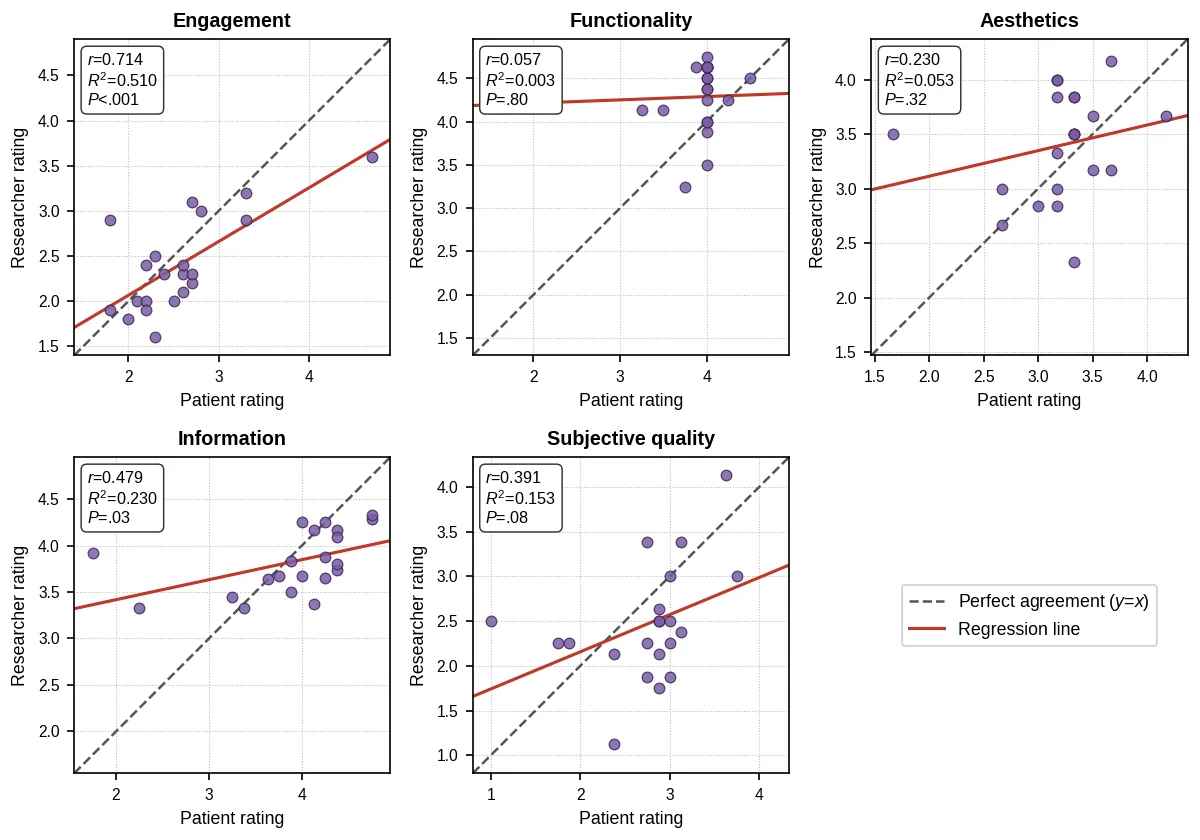
Dimension-level comparison of researcher and patient ratings.

### Association Structure Within Each Group

Scatter matrix analysis (Figure 3) revealed divergent evaluation frameworks. Patient ratings showed tight linear patterns, most notably for functionality-subjective quality (*r*=0.890; *P*<.001). In contrast, researcher ratings exhibited greater scatter for this relationship (*r*=0.489; *P*=.02, n=21; Δ*r*=0.401), indicating more compartmentalized assessment. Patient judgments were therefore more strongly organized around a single evaluative axis, while researcher judgments were more compartmentalized across dimensions. The bivariate relationships that differ most between the 2 groups are shown side by side in Figure 4.

**Figure 3:**
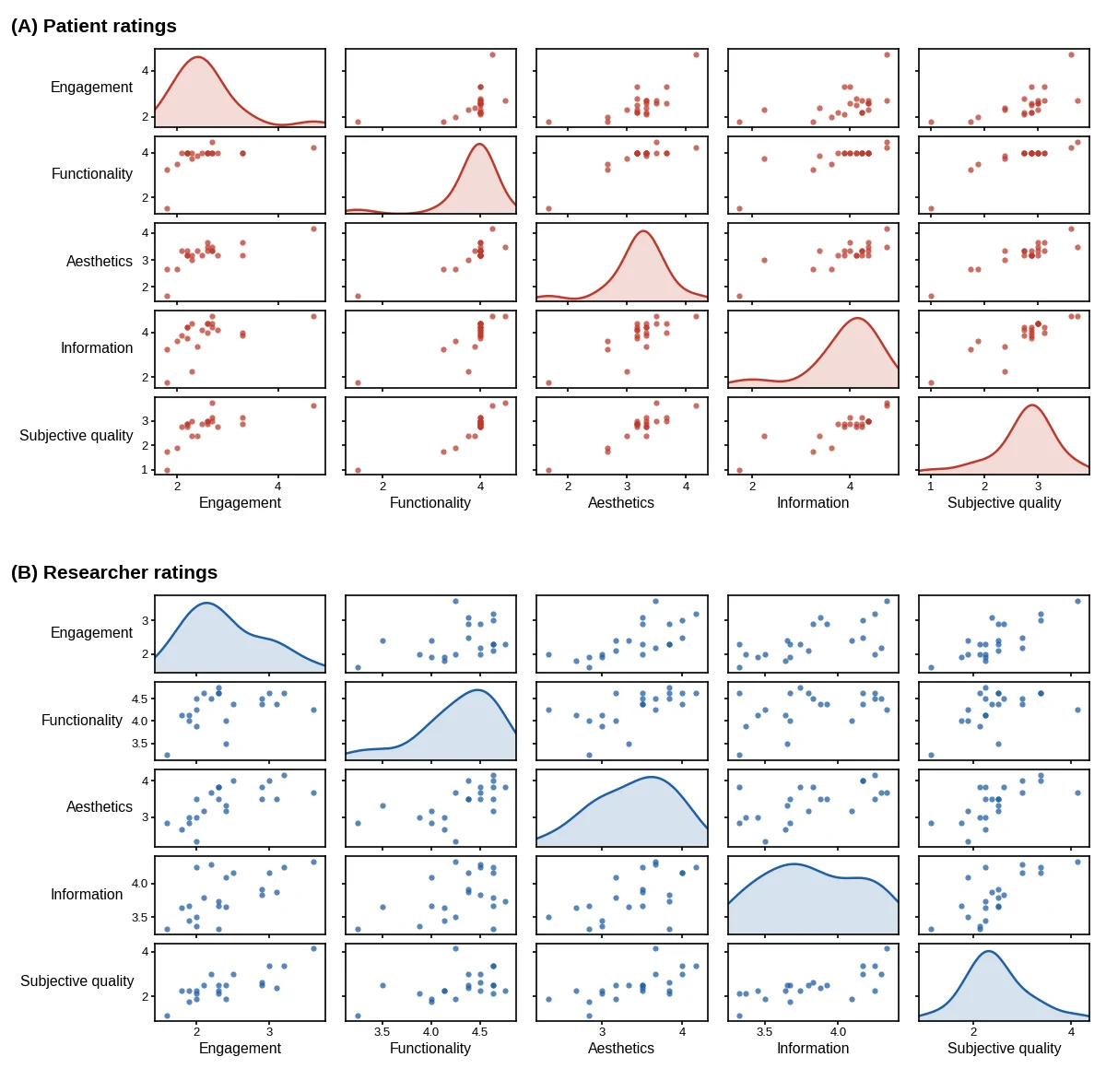
Scatter matrices for patient (A) and researcher (B) ratings.

**Figure 4:**
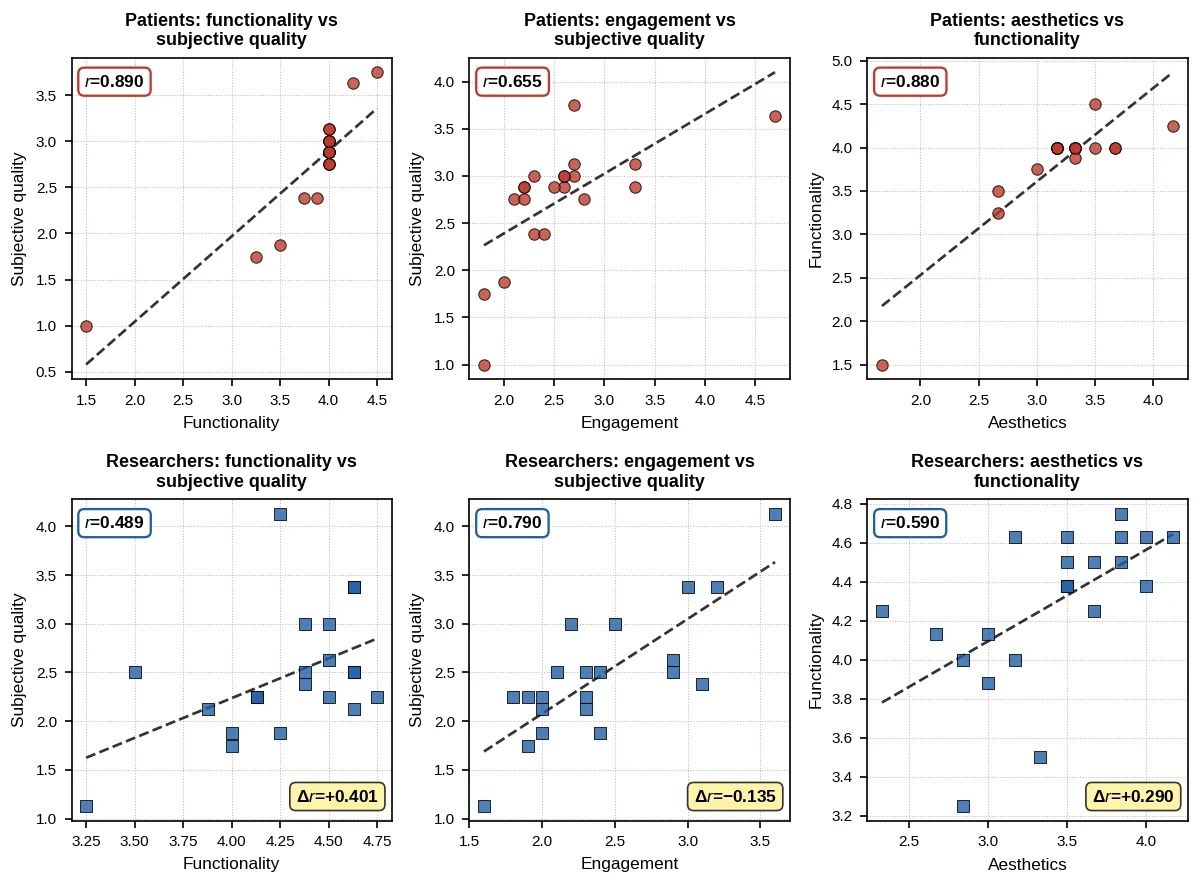
Key bivariate relationships compared between groups.

### Calibration

Calibration anchors differed between researcher and patient datasets, reflecting distinct rating distributions (Table 4). For researchers, the full membership threshold for engagement was 3.20, compared to 3.30 for patients. Functionality calibration showed more pronounced differences: researchers’ full membership threshold was 4.63 vs 4.25 for patients. Information quality thresholds demonstrated the greatest divergence: researchers set full membership at 4.29, whereas patients used 4.75. For the outcome variable (subjective quality), full membership thresholds were 3.38 for researchers and 3.63 for patients.

**Table 4:** Calibration anchors.

Variable	Type	R^a^ full^b^	R crossover	R nonmem.^c^	P^d^ full	P crossover	P nonmem.
Engagement	Continuous	3.20	2.30	1.80	3.30	2.50	1.80
Functionality	Continuous	4.63	4.38	3.50	4.25	4.00	3.25
Aesthetics	Continuous	4.00	3.50	2.67	3.67	3.33	2.67
Information	Continuous	4.29	3.80	3.33	4.75	4.13	2.25
Privacy	Binary	1	N/A^e^	0	1	N/A	0
Developer type	Binary	1	N/A	0	1	N/A	0
Subjective quality	Continuous	3.38	2.38	1.75	3.63	2.88	1.75

^a^R: researcher.

^b^full: full membership.

^c^nonmem.: full nonmembership.

^d^P: patient.

^e^N/A: not applicable.

These calibration differences underscore the importance of group-specific analysis in fsQCA, as identical raw scores may represent different levels of membership in the fuzzy set of “high quality” depending on the evaluator group’s reference frame. They should not be read as evidence that one group applied a stricter standard than the other in any absolute sense, since MARS and uMARS items are not scaled to a common metric.

### Necessary Conditions

Necessity analysis results are presented in Table 5 and Figure 5. For researcher evaluations, no single condition met the conventional threshold (consistency ≥0.90) for being considered necessary. Information quality demonstrated the highest necessity consistency (0.845), followed by functionality (0.821) and aesthetics (0.820). Engagement showed lower necessity (0.795), while privacy protection (0.322) and government developer type (0.242) were far below the threshold. Their negations showed moderate consistency, indicating that nongovernmental apps without formal privacy features could still receive high researcher ratings.

**Table 5:** Necessity analysis for high subjective quality.

Condition	R^a^ cons.^b^	R cov.^c^	R ~^d^cons.	R ~cov.	P^e^ cons.	P cov.	P ~cons.	P ~cov.
Engagement	0.795	0.807	0.490	0.460	0.829	0.844	0.594	0.547
Functionality	0.821	0.719	0.456	0.501	0.907	0.940	0.721	0.653
Aesthetics	0.820	0.815	0.506	0.484	0.841	0.870	0.627	0.568
Information	0.845	0.804	0.482	0.482	0.915	0.862	0.594	0.588
Privacy	0.322	0.660	0.678	0.434	0.269	0.546	0.731	0.463
Developer type	0.242	0.354	0.758	0.554	0.283	0.410	0.717	0.520

^a^R: researcher.

^b^cons.: consistency.

^c^cov.: coverage.

^d^~: negation of the condition.

^e^P: patient.

**Figure 5:**
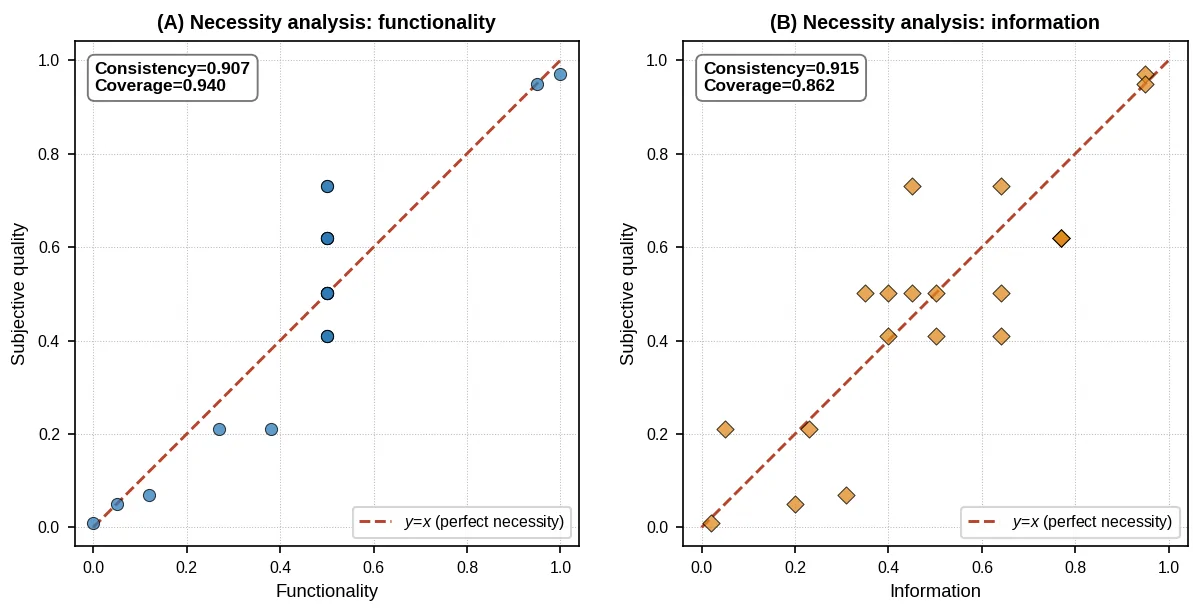
Necessity XY plots.

For patient evaluations, functionality (0.907) and information quality (0.915) both exceeded the threshold. However, these 2 conditions were highly correlated in the patient data (*r*=0.816), which means they cannot be treated as independent necessary conditions; a high correlation between candidate necessary conditions is a known source of overidentification in necessity analysis [67]. Following Schneider and Wagemann [67], we resolved this on substantive grounds: functionality is the precondition for reaching content at all, since a user must be able to navigate an app before its information can be judged, and information quality is best read as an extension of that access rather than a separate requirement. Functionality is therefore reported as the single candidate necessary condition for patients. We emphasize that with 21 cases and 1 panel, this should be treated as a proposition for testing rather than as an established necessity claim. Aesthetics (0.841) and engagement (0.829) approached but did not meet the threshold; privacy (0.269) and government developer type (0.283) did not.

### Sufficient Configurations: Researchers

Sufficiency analysis was conducted to identify configurational pathways leading to high subjective quality ratings. Five sufficient configurations were identified for high subjective quality ratings among researchers (Table 6). The overall solution consistency was 0.981, and solution coverage was 0.649. These values exceed the recommended threshold of 0.80 for consistency and indicate acceptable empirical coverage.

**Table 6:** Sufficient configurations for high subjective quality—researchers.

Configuration	Causal path	Consistency	Raw coverage	Unique coverage
R1	~^a^Developer × privacy × engagement × aesthetics × information	1.000	0.159	0.033
R2	~Developer × privacy × functionality × aesthetics × information	1.000	0.163	0.037
R3	~Developer × engagement × functionality × aesthetics × information	0.985	0.463	0.293
R4	Privacy × engagement × functionality × aesthetics × information	1.000	0.175	0.049
R5	~Developer × ~privacy × engagement × ~functionality × ~aesthetics × ~information	0.942	0.110	0.066
Solution	N/A^b^	0.981	0.649	N/A

^a^~: negation of the condition.

^b^N/A: not applicable.

Configuration R3 (nongovernment developer × high engagement × high functionality × high aesthetics × high information quality) demonstrated the highest raw coverage (0.463) and unique coverage (0.293). This pathway accounted for the largest proportion of high-quality outcomes. The consistency of 0.985 indicates that this configuration almost always leads to high subjective ratings.

Configurations R1 and R2 both combined privacy protection with aesthetics and information quality, each at consistency 1.000, differing only in whether engagement or functionality completed the combination. Within this dataset, the two therefore behave as substitutable elements once privacy is present, though with a single case supporting each pathway, this substitutability is a description of these cases rather than a generalizable finding.

Configuration R4 combined all positive conditions (privacy, engagement, functionality, aesthetics, and information) without specifying developer type. This comprehensive pathway achieved perfect consistency (1.000) with moderate coverage (0.175).

Configuration R5 is a contrarian pathway: nongovernmental apps without privacy features, with engagement but low functionality, aesthetics, and information quality (consistency 0.942, raw coverage 0.110). Only one app instantiates it, and it is reported for completeness rather than as a recommended design route.

### Sufficient Configurations: Patients

Four sufficient configurations emerged from patient evaluations (Table 7). The overall solution consistency was 0.998, and solution coverage was 0.796. Both metrics exceeded researcher evaluation results, indicating stronger explanatory power.

**Table 7:** Sufficient configurations for high subjective quality—patients.

Configuration	Causal path	Consistency	Raw coverage	Unique coverage
P1	~^a^Developer × ~privacy × engagement × functionality × aesthetics	1.000	0.285	0.018
P2	~Privacy × engagement × functionality × aesthetics × information	0.996	0.474	0.207
P3	~Developer × engagement × functionality × aesthetics × information	1.000	0.482	0.215
P4	~Developer × ~privacy × ~engagement × functionality × ~aesthetics × information	1.000	0.263	0.089
Solution	N/A^b^	0.998	0.796	N/A

^a^~: negation of the condition.

^b^N/A: not applicable.

Configuration P3 (nongovernment developer × high engagement × high functionality × high aesthetics × high information quality) demonstrated the highest coverage (raw=0.482, unique=0.215). This pathway closely mirrors researcher configuration R3, suggesting one shared route to a favorable judgment across groups.

Configuration P2 (absence of privacy × high engagement × high functionality × high aesthetics × high information quality) achieved near-perfect consistency (0.996) with substantial coverage (0.474). The appearance of privacy absence as a component distinguishes patient from researcher solutions, but it should be read against the distribution of the condition: 16 of 21 apps lacked privacy protection, so most apps that were strong on the 4 MARS dimensions were also, necessarily, apps without it. It does not indicate that the patient panel rated protected apps lower. In set-theoretic terms, protected apps were slightly more consistently members of the high-rating set than unprotected ones (0.546 vs 0.463; Table 5), and the 2 apps the patient panel rated highest, Chabei and Hong Feng Wan, both offered protection.

Configuration P1 emphasized engagement, functionality, and aesthetics without requiring high information quality. This pathway indicates that visually appealing and functionally robust apps can achieve high patient ratings even with moderate information content.

Configuration P4 is compensatory: apps lacking engagement and aesthetics still achieved high ratings through functionality and information quality (consistency 1.000, raw coverage 0.263).

### Comparison Across Groups

Table 8 presents a consolidated comparison of configurations across evaluator groups. Several patterns warrant attention.

**Table 8:** Configurational comparison—researchers and patients.

Antecedent condition	R1	R2	R3	R4	R5	P1	P2	P3	P4
Engagement	✓^a^	—^b^	✓	✓	✓	✓	✓	✓	X^c^
Functionality	—	(✓)^d^	(✓)	(✓)	X	(✓)	(✓)	(✓)	✓
Aesthetics	✓	✓	✓	✓	(X)^e^	✓	✓	✓	X
Information	✓	✓	✓	✓	(X)	—	✓	✓	✓
Privacy	✓	✓	—	✓	(X)	(X)	(X)	—	(X)
Developer type	X	X	X	—	X	X	—	X	X
Consistency	1.000	1.000	0.985	1.000	0.942	1.000	0.996	1.000	1.000
Raw coverage	0.159	0.163	0.463	0.175	0.110	0.285	0.474	0.482	0.263
Unique coverage	0.033	0.037	0.293	0.049	0.066	0.018	0.207	0.215	0.089
Solution consistency	0.981	N/A^f^	N/A	N/A	N/A	0.998	N/A	N/A	N/A
Solution coverage	0.649	N/A	N/A	N/A	N/A	0.796	N/A	N/A	N/A

^a^✓: core condition present.

^b^Em dashes indicate that the condition may be either present or absent.

^c^X: core condition absent.

^d^(✓): peripheral condition present.

^e^(X): peripheral condition absent.

^f^N/A: not applicable.

First, functionality appeared as a core or peripheral condition in most configurations for both groups. For patients, functionality was present in all 4 configurations. For researchers, functionality appeared in 3 of 5 configurations. This finding aligns with necessity analysis results showing functionality as highly important for both groups.

Second, privacy protection plays different roles. It is a core condition in 3 researcher configurations, whereas its negation appears in 3 of 4 patient configurations. Since privacy protection was present in only 5 of 21 apps, both patterns rest on few cases. The contrast is a difference in configurational role, not a difference in valuation: the negated condition enters the patient solution because unprotected apps dominate the sample, and protected apps were, if anything, rated slightly more consistently highly by the patient panel (Table 5). The skewed distribution of this condition is a limitation of the sample rather than a finding about the evaluators.

Third, engagement carried different weight in the 2 panels. Three of 4 patient configurations included engagement as a core condition, and in 2 of those it is one of only 3 core conditions. For researchers, engagement was a core condition in 4 of 5 configurations, but in 3 of those it appeared alongside 3 or 4 further core conditions.

Fourth, nongovernmental development appears across both groups’ high-performing configurations. Because government-developed apps in this sample were predominantly county-level CDC applets with limited interactive design, this condition plausibly indexes development resources and design intent rather than ownership as such.

Fifth, solution coverage differed substantially between groups (researchers: 0.649; patients: 0.796). The higher patient coverage indicates that the identified configurations account for a larger proportion of the outcomes rated highly by the 2 patient evaluators.

### Robustness and Sensitivity Analyses

#### Calibration Anchors

Recomputing every configuration under 4 anchor sets left the configurational structure unchanged (Table 9). Across all anchor sets, every configuration retained consistency at or above 0.942, and solution-level values remained high (researchers: consistency 0.966-0.983, coverage 0.639-0.679; patients: consistency 0.997-1.000, coverage 0.582-0.796). The baseline column of Table 9 is the set of values reported in Tables 6 and 7. Coverage was more anchor-sensitive than consistency, and most so for the patient configurations, which is expected because relaxing the full-membership anchor rescales membership scores in the outcome.

**Table 9:** Sensitivity of configuration consistency and raw coverage to calibration anchors^a^.

Config.^b^	Cons.^c^ 95/50/5	Cons. 90/50/10	Cons. 85/50/15	Cons. 80/50/20	Cov.^d^ 95/50/5	Cov. 90/50/10	Cov. 85/50/15	Cov. 80/50/20
R1	1.000	1.000	1.000	1.000	0.159	0.159	0.159	0.167
R2	1.000	1.000	1.000	1.000	0.163	0.159	0.152	0.166
R3	0.985	0.982	0.987	0.959	0.463	0.454	0.422	0.442
R4	1.000	1.000	1.000	1.000	0.175	0.167	0.154	0.162
R5	0.942	0.948	0.944	0.944	0.110	0.103	0.084	0.082
P1	1.000	1.000	1.000	1.000	0.285	0.233	0.182	0.111
P2	0.996	1.000	1.000	1.000	0.474	0.359	0.316	0.219
P3	1.000	1.000	1.000	1.000	0.482	0.397	0.384	0.306
P4	1.000	1.000	1.000	1.000	0.263	0.219	0.181	0.153

^a^Values were recomputed from the calibrated data using identical procedures; the 95/50/5 column reproduces Tables 6 and 7.

^b^Config.: configuration.

^c^Cons.: consistency.

^d^Cov.: coverage.

#### Necessity Across Anchors

Necessity consistencies were recomputed under each anchor set. For researchers, no condition reached 0.90 under any specification, so the conclusion that no single condition is necessary for researcher judgments is not an artifact of the anchors. For patients, functionality (0.907) and information quality (0.915) exceeded the threshold at the baseline anchors only; under the three wider sets neither reached it (functionality 0.744-0.804; information quality 0.824-0.860). Two features of the patient data account for this. First, patient functionality ratings are concentrated at 4.00, so that the 80th, 85th, and 90th percentiles coincide with the crossover, and the full-membership anchor had to be set to the nearest observed value beyond it. Second, widening the anchors compresses membership scores toward the crossover and thereby lowers necessity consistency for conditions whose upper values are closely spaced. The patient necessity result is therefore anchor-dependent, which reinforces rather than qualifies the caution stated in the Necessary Conditions section: functionality is offered as a proposition for testing and not as an established necessary condition. Privacy protection remained far below threshold in every specification for both groups (0.264-0.343). The full necessity sensitivity results are given in Table 10.

**Table 10:** Necessity consistency for high subjective quality under 4 calibration anchor sets^a^.

Group	Condition	95/50/5	90/50/10	85/50/15	80/50/20
Researchers	Engagement	0.795	0.787	0.767	0.783
Researchers	Functionality	0.821	0.780	0.738	0.756
Researchers	Aesthetics	0.820	0.809	0.806	0.811
Researchers	Information	0.845	0.850	0.804	0.839
Researchers	Privacy	0.322	0.329	0.326	0.343
Researchers	Developer type	0.242	0.236	0.238	0.226
Patients	Engagement	0.829	0.730	0.788	0.765
Patients	Functionality	0.907	0.804	0.795	0.744
Patients	Aesthetics	0.841	0.805	0.738	0.645
Patients	Information	0.915	0.849	0.860	0.824
Patients	Privacy	0.269	0.266	0.277	0.264
Patients	Developer type	0.283	0.284	0.279	0.288

^a^Values are necessity consistencies. The conventional threshold is 0.90. Column headings give the percentile anchors used for full membership, crossover, and full nonmembership.

#### Consistency and PRI Thresholds

Fourteen of the 64 truth table rows were empirically instantiated in the researcher data and 15 in the patient data. Under the baseline cutoffs, 6 researcher rows and 5 patient rows met both criteria. Across the 16 combinations of raw consistency cutoffs (0.75, 0.80, 0.85, 0.90) and PRI cutoffs (0.65, 0.70, 0.75, 0.80), the retained set was invariant in the patient data. In the researcher data, it was invariant with respect to the raw consistency cutoff, but the row instantiated by the Beijing AIDS Association applet, whose PRI consistency is 0.712, dropped out at PRI cutoffs of 0.75 and above, which removes configuration R5 from the solution. R5 is the contrarian pathway, is instantiated by that single case, and has the lowest consistency and the lowest raw coverage of the configurations reported; the other 4 researcher configurations were unaffected at every combination tested. The solutions are therefore insensitive to the raw consistency cutoff, and sensitive to the PRI cutoff only in respect of R5.

#### Alternative Privacy Operationalization

Re-running the analysis with the 3-level privacy set left every configuration unchanged in substance: the truth tables retained the same rows, Boolean minimization returned the same 5 researcher and 4 patient configurations, and the parsimonious solutions were identical. Consistency shifted by at most 0.018 (patient P4: 1.000 to 0.982), and no configuration fell below 0.947. Coverage changes were likewise small, the largest being increases of 0.052 in the raw coverage of patient P1 (0.285 to 0.337) and of 0.051 in that of P2 (0.474 to 0.525). Necessity consistency for privacy protection remained low under the graded specification (researchers 0.278; patients 0.223). The limited role of privacy in the patient configurations is therefore not an artifact of dichotomization: only 5 apps offered protection of any kind, only 2 of those added an in-app privacy password, and grading the 5 does not alter how privacy combines with the other conditions.

### Case-Level Analysis

XY plots were constructed to visualize the cases instantiating the highest-coverage configurations, R3 and P3 (Figure 6). All illustrative cases demonstrated configuration membership scores above 0.5 with corresponding outcome membership scores above 0.5, confirming sufficiency at the case level. No deviant cases were identified.

**Figure 6:**
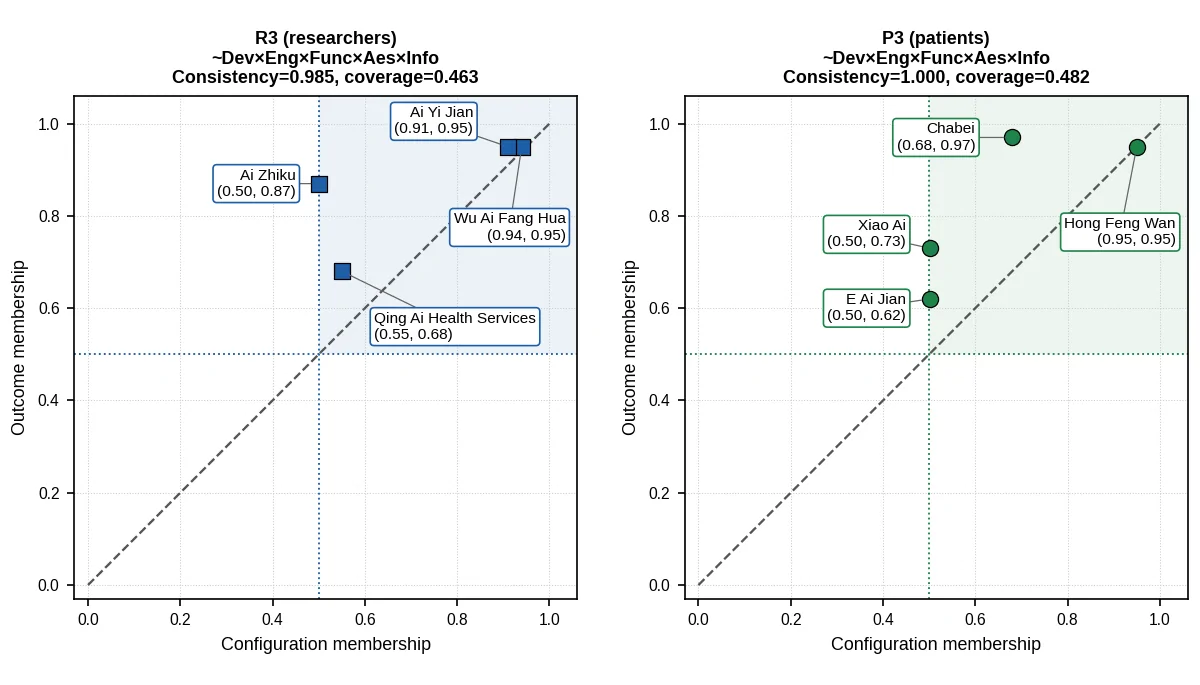
XY plots for case-level analysis of configurations R3 (researchers) and P3 (patients). Both configurations share the same structure (~Dev×Eng×Func×Aes×Info) and are the highest-coverage pathway for each evaluator group. Each point is an app; the values in parentheses are configuration membership and outcome membership, and values shown as 0.50 are 0.501 after the adjustment described in the Calibration section. Aes: aesthetics; Dev: government developer; Eng: engagement; Func: functionality; Info: information quality; ~: negation of the condition.

For researchers, configuration R3 contained the most cases (n=4), including Wu Ai Fang Hua (0.94, 0.95) and Ai Yi Jian (0.91, 0.95). For patients, configuration P3 was best exemplified by Chabei (0.68, 0.97).

R3 and P3 share an identical structure (~developer [~ denotes negation of the condition] × engagement × functionality × aesthetics × information), yet different apps sit at the top of each. This indicates that the same combination of dimensions is satisfied by different apps depending on who is rating. These apps are presented as illustrations of each pathway, not as validated exemplars: with 1 to 4 cases per configuration, they show what a configuration looks like in practice and support interpretation, but they do not constitute evidence that these are the best available apps.

## Discussion

### Principal Findings

Configurational pathways to high subjective quality in HIV/AIDS apps were examined using fsQCA. Three key findings emerged. First, equifinality was demonstrated: 5 configurations were identified for researchers, and 4 for patients, and no single pathway was required for high-quality ratings. Second, the 2 panels’ evaluation priorities differed in this sample. Privacy protection was a core condition in 3 researcher configurations and appeared as a present condition in none of the patient ones, whose configurations instead combined engagement, functionality, aesthetics, and information quality with the absence of privacy protection. Third, functionality emerged as foundational: it reached the necessity threshold in the patient data and appeared in most configurations for researchers. Wilcoxon tests independently confirmed that functionality is where the 2 groups diverge most in level and agree least in rank order.

These findings are broadly consistent with the theoretical framework outlined earlier, with one important qualification. The prominence of functionality and engagement in patient configurations matches the task-technology fit and performance-expectancy predictions [20-22]: for a person managing HIV, an app earns a favorable judgment by letting them accomplish something. The privacy calculus prediction that end users discount abstract, delayed risks [23,24] is not, however, supported by these data. Privacy protection was absent from the patient pathways because it was absent from most apps, not because protected apps were rated lower; the 2 apps the patient panel rated highest both offered protection. What the data do show is that protection was not required for a favorable patient judgment, which is consistent with the eHealth literacy account that such features are hard for end users to appraise [25], but equally consistent with users valuing protection when it is present. The compartmentalized structure of researcher ratings, in contrast, is what one would expect from trained raters applying a normative checklist rather than pursuing a task.

### Interpreting the Divergence

Three nonexclusive mechanisms may explain the divergence, and they carry different implications.

If the mechanism is appraisal capacity—privacy features are simply not visible or assessable to end users [25,69]—the appropriate response is disclosure design: making protective features legible at the point of use, as nutrition labeling does for food.

If the mechanism is privacy calculus—users perceive the features but discount them against immediate benefit [23,24]—disclosure alone will not change judgments, and the appropriate response is to reduce the cost of the protective option rather than to inform users about it.

If the mechanism is displaced trust—users infer safety from health-service branding or from CDC association—then the risk is concentrated precisely in the apps that appear most institutionally credible, and the appropriate response is certification rather than user education.

The present data cannot distinguish these mechanisms, because no qualitative data on evaluator reasoning were collected. This is a limitation of the design and the most important priority for follow-up work: think-aloud protocols or postrating interviews with a larger patient panel would discriminate among the three, and the practical recommendation differs substantially depending on which holds.

### Illustrative Cases and Design Implications

Case-level analysis identifies apps that instantiate each pathway and, more usefully for developers, the concrete features they share; these features are the level at which user-centered design guidance for HIV apps has previously been formulated [79].

Wu Ai Fang Hua and Ai Yi Jian illustrate the researcher-oriented pathway R3. Common features include a structured knowledge base with attributed sources and update dates, medication and follow-up reminders, and a web-based community component. The design lesson is not “score highly on four dimensions,” but that content credibility signals—visible sourcing, dating, and institutional attribution—are what distinguish these apps from otherwise similar applets.

Chabei illustrates the patient-oriented pathway P3. Its distinguishing features are a shallow navigation hierarchy in which testing appointment booking is reachable in 2 taps from launch, plain-language content without clinical jargon, and a peer community area. Its high outcome membership (0.97) relative to its configuration membership (0.68) suggests that features outside the MARS/uMARS subscales contribute to patient satisfaction—most plausibly the anonymity and low-friction access that a WeChat applet affords, since no download, registration, or app-store trace is required.

Configuration P4 is the practically relevant pathway for resource-constrained developers: high ratings from this patient panel were achieved through functionality and information quality alone, without investment in engagement or visual design. For county-level CDC teams building service applets on limited budgets, this suggests that reliable booking and accurate, current content are the necessary investments, and that gamified or visually elaborate features are optional rather than foundational.

Conversely, configuration R5 contained a single marginal case (Beijing AIDS Association: 0.58, 0.59) whose recognition rested on engagement despite low scores elsewhere, a reminder that expert endorsement does not transfer automatically to user acceptance.

### Recommendations for Developers

#### Overview

The configurations and the illustrative cases translate into a small number of concrete design decisions. They are stated below at feature level, because that is the level at which a development team acts, and recommendations that follow directly from the observed cases are distinguished from those that are proposals consistent with the findings but not themselves tested here. Table 11 sets each against the configuration from which it derives.

**Table 11:** Feature-level design recommendations derived from the observed configurations and illustrative cases^a^.

Configuration (panel)	Illustrative cases	Observed feature pattern	Recommendation	Evidential status
P3, P4 (patients)	Chabei	Core service task 2 taps from launch; content readable without registration	Keep booking, reminders, and result checking within 2 taps; allow anonymous reading	Follows from observed cases
R3 (researchers)	Wu Ai Fang Hua; Ai Yi Jian	Attributed and dated knowledge base; reminders; community area	Show source, date, and reviewing institution on every content item; keep a visible update log	Follows from observed cases
R1, R2, R4 (researchers) vs P1-P4 (patients)	—^b^	Privacy protection core in 3 researcher configurations; present in none of the patient configurations	Enable protection by default; keep notification text free of HIV-related content; summarize storage on one screen at first launch	Proposal consistent with the finding
P4 (patients)	—	High ratings from functionality and information quality alone	Fund booking reliability and content currency first; treat engagement and visual design as later increments	Follows from observed cases
R5 (researchers)	Beijing AIDS Association	Engagement present, other dimensions low; single marginal case	Do not treat engagement as a substitute for the service task: this route appeared only in the researcher solution	Single marginal case; researcher side only
P3 (patients)	Chabei	Outcome membership (0.97) above configuration membership (0.68); applet format	Consider applet delivery where feasible, treating the absence of download, icon, and store record as a privacy feature	Interpretation; not directly measured
Across panels	—	Divergence between panels in the appraisal of privacy protection	Prefer platform- or CDC-level privacy certification to user-facing privacy education	Policy proposal consistent with the finding

^a^Recommendations marked as proposals are design implications consistent with the observed configurations but not themselves tested in this study. Configuration labels follow Table 8.

^b^Not available.

#### Keep the Primary Service Task Within 2 Taps of Launch

Functionality entered all 4 patient configurations and 3 of the 5 researcher ones, and it is the condition retained as the candidate necessary condition for the patient panel; Chabei, the illustrative case for the patient pathway P3, placed testing appointment booking 2 taps from launch. Appointment booking, reminder setting, and result checking should not sit behind a menu tree, a splash sequence, or a sign-in screen.

#### Do Not Require Registration to Read Information

Information quality entered every patient configuration except P1, and registration is the point at which an identifiable record is created. The recruitment experience reported in Methods indicates how heavily that possibility weighs on this population. Content should be readable anonymously, with account creation required only for functions that cannot operate without it.

#### Make Content Provenance Visible on Every Item

The 2 apps illustrating R3, Wu Ai Fang Hua and Ai Yi Jian, were distinguished from otherwise similar applets by a knowledge base whose entries carried attributed sources and update dates. Each content item should therefore name its source, carry a publication or revision date and, where applicable, identify the institution that reviewed it, with an in-app record of when the content set was last updated as a whole.

#### Treat Protective Settings as Defaults Rather Than as Options

Privacy protection was a core condition in 3 researcher configurations and appeared as a present condition in none of the patient ones. Whatever the mechanism, a design that requires the user to seek protection out will under-protect this population. Proposals consistent with this finding, though not tested in this study, are to enable any available protection by default rather than on request, to write notification and reminder text that never names HIV status or medication, and to present what is stored and where on a single plain-language screen at first launch rather than within a terms-of-use document.

#### Fund Function and Content Before Engagement and Visual Design

Configuration P4 reached high ratings in the patient panel on functionality and information quality alone. For a county-level team, this makes booking reliability and content currency the first 2 line items and gamified or visually elaborate features later increments. Configuration R5 makes a related point from the other side. One app reached a favorable researcher judgment on engagement alone, with functionality, aesthetics, and information quality all low; but that route appeared only in the researcher solution, rests on a single case, and has the lowest consistency and the lowest raw coverage of any configuration reported. Engagement can carry an expert’s overall impression of an app, and on the evidence here it does not stand in for the service task on the user side.

#### Consider an Applet Build Where the Service Permits It

The outcome membership for Chabei (0.97) exceeded its configuration membership (0.68), which suggests a contribution from something outside the rated dimensions, most plausibly that a WeChat applet requires no download, leaves no icon on the home screen, and creates no app-store record. Where the service can be delivered in that format, the distribution format is itself a privacy feature. This reading is an interpretation of a single case rather than a measured effect.

#### Prefer Certification to User-Facing Privacy Education

Because privacy protection did not appear as a present condition in any patient pathway, publishing more privacy information is unlikely on its own to alter user-side judgments. A visible privacy label issued at platform or CDC level would place the appraisal burden where the capacity to carry it lies. This is a policy proposal consistent with the observed divergence rather than a finding of the study.

### Comparison With Prior Research

Prior MARS-based work has documented correlational differences between expert and user ratings [35,80] and has evaluated HIV/AIDS apps against mean-score benchmarks [36-38]. The present study extends this literature by describing how each group’s judgments are organized rather than only whether they differ: the same dimensions enter different combinations for different evaluators.

The centrality of functionality is consistent with usability findings across health app domains, including behavioral intervention technologies [81] and mental health apps [82]. This supports the interpretation that functional performance serves as a baseline requirement rather than a differentiator.

The privacy finding aligns with the broader digital health literature documenting weak privacy practice and limited user appraisal of it [68-71,83], and with the privacy paradox [23,24]. For HIV/AIDS populations, the stakes are higher than in most app categories, since disclosure carries risks of stigma, relationship loss, and discrimination [18,19].

### Limitations

#### Overview

Several limitations bound the claims that can be made from these data.

#### Patient Panel Size

The patient perspective rests on 2 evaluators whose ratings were averaged. Although a 2-person panel is consistent with the evaluator conventions of this literature (Table 1), and although both evaluators completed the full protocol for all 21 apps with narrow limits of agreement, 2 end users cannot capture the heterogeneity of people living with HIV in China. It is therefore not possible to determine from these data whether the patient configurations reflect preferences shared across this population or the perspectives of these 2 individuals. The patient findings are accordingly presented as exploratory and hypothesis-generating: the claim supported by these data is that a configurational logic distinct from the expert one is observable, not that it is the logic of the patient population.

#### Case Number and Conditions

Twenty-one apps with 6 conditions produce pronounced limited diversity: only 14 of 64 logically possible configurations (researcher data) and 15 of 64 (patient data) contained empirical cases. Several configurations rest on one or two apps, and unique coverage values below 0.05 (R1, R2, R4, and P1) indicate pathways with little independent empirical weight. The sensitivity analyses show the solutions are largely stable under alternative analytic choices, with the exceptions reported in the Robustness and Sensitivity Analyses section of the Results, but stability under respecification is not the same as generalizability to other app populations.

#### Rater Panel Composition

Researcher ratings came from 3 individuals from 2 institutions; although interrater agreement was close, evaluator effects cannot be excluded [26], and a differently composed expert panel might weight dimensions differently.

#### Absence of Qualitative Data

No interview or think-aloud data were collected, so the mechanisms proposed for the observed divergence—appraisal capacity, privacy calculus, displaced trust—are interpretations consistent with theory rather than findings.

#### Privacy Measurement

Even under the graded specification, privacy was assessed from user-facing artifacts. Actual data handling, third-party sharing, and permission behavior were not examined, and apps that display a policy may not comply with it [70,71].

#### External Validation

Quality was operationalized as rated subjective quality. No downstream outcome—sustained use, adherence, testing uptake, or clinical benefit—was measured, so the configurations describe pathways to a favorable evaluation, not to health impact.

#### Context and Time

Only Simplified Chinese apps available in early 2023 were examined. The market changes quickly, WeChat applets are a China-specific format, and quality drivers may differ in other linguistic and regulatory settings.

### Future Research Directions

Priorities follow directly from the limitations. Recruiting a substantially larger and more diverse patient panel, ideally stratified by age, sex, time since diagnosis, and eHealth literacy, would allow the patient configurations reported here to be tested rather than proposed. Adding qualitative data would discriminate among the mechanisms proposed for the privacy divergence. Extending the analysis to a larger app corpus, or pooling across countries, would reduce limited diversity and permit conditions to be added without exceeding what the case number supports. Linking configurational membership to downstream use and clinical outcomes would establish whether pathways to high perceived quality are also pathways to benefit. Finally, experimental manipulation of specific features would move the design implications from association toward causal evidence.

### Conclusions

Researcher and patient evaluations of Chinese HIV/AIDS mobile apps followed different configurational logics in this exploratory study. Privacy protection entered only the researcher pathways to high subjective quality, whereas the patient panel’s pathways combined engagement, functionality, aesthetics, and information quality with its absence; functionality entered all 4 patient configurations and 3 of the 5 researcher ones, and met the necessity threshold in the patient data at the baseline calibration. Multiple equifinal pathways were identified in each group, so no single feature combination is required for a favorable evaluation.

These patterns were largely stable across calibration anchors, consistency thresholds, and an alternative graded operationalization of privacy; the exceptions are that 1 researcher configuration did not survive the stricter PRI cutoffs and that the patient necessity result held only at the baseline anchors. They rest, however, on 21 apps rated by 3 researchers and 2 patients, and the patient findings in particular should be read as hypotheses about how end users form quality judgments rather than as estimates of what people living with HIV prefer.

Two implications are nonetheless warranted. First, expert and end user assessments are not interchangeable, and app recommendation and development processes that rely on either alone risk producing apps that are respectively unused or underprotected. Second, privacy protection was rare in this market and was not required for a favorable patient judgment, although the apps the patient panel rated highest did offer it. In a population for which the cost of unrecognized disclosure risk is not measured in inconvenience, protection therefore needs to be ensured by developers and regulators rather than left to user demand.

## Data Availability

The datasets used and analyzed during the current study are available from the corresponding author upon reasonable request.

## Abbreviations

CDC: Center for Disease Control and Prevention
fsQCA: fuzzy-set qualitative comparative analysis
ICC: intraclass correlation coefficient
MARS: Mobile App Rating Scale
mHealth: mobile health
PRI: proportional reduction in inconsistency
PRISMA: Preferred Reporting Items for Systematic Reviews and Meta-Analyses
QCA: qualitative comparative analysis
uMARS: user version of Mobile App Rating Scale
UTAUT: unified theory of acceptance and use of technology
UTAUT2: extended unified theory of acceptance and use of technology

## References

[1] Joint United Nations Programme on HIV/AIDS. Global HIV & AIDS statistics — fact sheet. UNAIDS.

[2] Muessig KE, Nekkanti M, Bauermeister J, Bull S, Hightow-Weidman LB (2015). A systematic review of recent smartphone, internet and Web 2.0 interventions to address the HIV continuum of care. Curr HIV/AIDS Rep.

[3] Schnall R, Travers J, Rojas M, Carballo-Diéguez A (2014). eHealth interventions for HIV prevention in high-risk men who have sex with men: a systematic review. J Med Internet Res.

[4] Finitsis DJ, Pellowski JA, Johnson BT (2014). Text message intervention designs to promote adherence to antiretroviral therapy (ART): a meta-analysis of randomized controlled trials. PLoS One.

[5] Hamine S, Gerth-Guyette E, Faulx D, Green BB, Ginsburg AS (2015). Impact of mHealth chronic disease management on treatment adherence and patient outcomes: a systematic review. J Med Internet Res.

[6] Krebs P, Duncan DT (2015). Health app use among US mobile phone owners: a national survey. JMIR mHealth uHealth.

[7] Payne HE, Lister C, West JH, Bernhardt JM (2015). Behavioral functionality of mobile apps in health interventions: a systematic review of the literature. JMIR mHealth uHealth.

[8] Grundy QH, Wang Z, Bero LA (2016). Challenges in assessing mobile health app quality: a systematic review of prevalent and innovative methods. Am J Prev Med.

[9] Baumel A, Muench F, Edan S, Kane JM (2019). Objective user engagement with mental health apps: systematic search and panel-based usage analysis. J Med Internet Res.

[10] Vaghefi I, Tulu B (2019). The continued use of mobile health apps: insights from a longitudinal study. JMIR mHealth uHealth.

[11] Nouri R, Kalhori SRN, Ghazisaeedi M, Marchand G, Yasini M (2018). Criteria for assessing the quality of mHealth apps: a systematic review. J Am Med Inf Assoc.

[12] Hutton L, Price BA, Kelly R, McCormick C, Bandara AK, Hatzakis T, Meadows M, Nuseibeh B (2018). Assessing the privacy of mHealth apps for self-tracking: heuristic evaluation approach. JMIR mHealth uHealth.

[13] Bol N, Helberger N, Weert JCM (2018). Differences in mobile health app use: a source of new digital inequalities?. Inf Soc.

[14] Perski O, Blandford A, West R, Michie S (2017). Conceptualising engagement with digital behaviour change interventions: a systematic review using principles from critical interpretive synthesis. Transl Behav Med.

[15] Stoyanov SR, Hides L, Kavanagh DJ, Zelenko O, Tjondronegoro D, Mani M (2015). Mobile App Rating Scale: a new tool for assessing the quality of health mobile apps. JMIR mHealth uHealth.

[16] LeBeau K, Huey LG, Hart M (2019). Assessing the quality of mobile apps used by occupational therapists: evaluation using the user version of the Mobile Application Rating Scale. JMIR mHealth uHealth.

[17] Stoyanov SR, Hides L, Kavanagh DJ, Wilson H (2016). Development and validation of the user version of the Mobile Application Rating Scale (uMARS). JMIR mHealth uHealth.

[18] Gillette E, Naanyu V, Nyandiko W, Chory A, Scanlon M, Aluoch J, Koros H, Ashimosi C, Beigon W, Munyoro D, Lidweye J, Nyagaya J, DeLong A, Kantor R, Vreeman R (2023). HIV-related stigma shapes research participation for youth living with HIV in Kenya. J Int Assoc Providers AIDS Care (JIAPAC).

[19] Earnshaw VA, Chaudoir SR (2009). From conceptualizing to measuring HIV stigma: a review of HIV stigma mechanism measures. AIDS Behav.

[20] Goodhue DL, Thompson RL (1995). Task-technology fit and individual performance. MIS Q.

[21] Venkatesh V, Morris MG, Davis GB, Davis FD (2003). User acceptance of information technology: toward a unified view. MIS Q.

[22] Venkatesh V, Thong JYL, Xu X (2012). Consumer acceptance and use of information technology: extending the unified theory of acceptance and use of technology. MIS Q.

[23] Dinev T, Hart P (2006). An extended privacy calculus model for e-commerce transactions. Inf Syst Res.

[24] Barth S, de Jong MDT (2017). The privacy paradox–investigating discrepancies between expressed privacy concerns and actual online behavior–a systematic literature review. Telematics Inf.

[25] Norman CD, Skinner HA (2006). eHEALS: the eHealth Literacy Scale. J Med Internet Res.

[26] Hertzum M, Jacobsen NE (2001). The evaluator effect: a chilling fact about usability evaluation methods. Int J Hum Comput Interact.

[27] Woodside AG (2013). Moving beyond multiple regression analysis to algorithms: calling for adoption of a paradigm shift from symmetric to asymmetric thinking in data analysis and crafting theory. J Bus Res.

[28] Misangyi VF, Greckhamer T, Furnari S, Fiss PC, Crilly D, Aguilera R (2017). Embracing causal complexity: the emergence of a neo-configurational perspective. J Manage.

[29] Greckhamer T, Misangyi VF, Elms H, Lacey R (2008). Using qualitative comparative analysis in strategic management research. Organ Res Methods.

[30] Ragin CC (2000). Fuzzy-Set Social Science.

[31] Ragin CC, Rihoux B, Ragin CC (2009). Qualitative comparative analysis using fuzzy sets (fsQCA). Configurational Comparative Methods: Qualitative Comparative Analysis (QCA) and Related Techniques.

[32] Thomas J, O'Mara-Eves A, Brunton G (2014). Using qualitative comparative analysis (QCA) in systematic reviews of complex interventions: a worked example. Syst Rev.

[33] Singh K, Drouin K, Newmark LP, Rozenblum R, Lee J, Landman A, Pabo E, Klinger EV, Bates DW (2016). Developing a framework for evaluating the patient engagement, quality, and safety of mobile health applications. Issue Brief (Commonw Fund).

[34] Terhorst Y, Rathner EM, Baumeister H, Sander LB (2018). ["Help from the app store?": a systematic review of depression apps in German app stores] [Article in German]. Verhaltenstherapie.

[35] Liu P, Wang L, Wang F (2024). Evaluation of Chinese HIV mobile apps by researchers and patients with HIV: quality evaluation study. JMIR mHealth uHealth.

[36] Fernández-Sánchez H, Salazar-Alberto J, Fernández-Delgado JCM, Galvin A, Mugavero MJ, Rodriguez-Diaz CE, Santa Maria D (2025). Mobile apps for HIV and sexually transmitted infection prevention in Canada, Mexico, and the United States: environmental scan. JMIR mHealth uHealth.

[37] Raeesi A, Khajouei R, Ahmadian L (2022). Evaluating and rating HIV/AIDS mobile apps using the feature-based application rating method and mobile app rating scale. BMC Med Inf Decis Making.

[38] Raeesi A, Khajouei R, Ahmadian L (2021). Evaluation of HIV/AIDS-related mobile health applications content using an evidence-based content rating tool. BMC Med Inf Decis Making.

[39] Yang G, Long J, Luo D, Xiao S, Kaminga AC (2019). The characteristics and quality of mobile phone apps targeted at men who have sex with men in china: a window of opportunity for health information dissemination?. JMIR mHealth uHealth.

[40] Pellowski JA, Kalichman SC, Matthews KA, Adler N (2013). A pandemic of the poor: social disadvantage and the U.S. HIV epidemic. Am Psychol.

[41] Page MJ, McKenzie JE, Bossuyt PM, Boutron I, Hoffmann TC, Mulrow CD, Shamseer L, Tetzlaff JM, Akl EA, Brennan SE, Chou R, Glanville J, Grimshaw JM, Hróbjartsson A, Lalu MM, Li T, Loder EW, Mayo-Wilson E, McDonald S, McGuinness LA, Stewart LA, Thomas J, Tricco AC, Welch VA, Whiting P, Moher D (2021). The PRISMA 2020 statement: an updated guideline for reporting systematic reviews. BMJ.

[42] National Health Commission, Ministry of Education, Ministry of Science and Technology, National Administration of Traditional Chinese Medicine Measures for Ethical Review of Life Science and Medical Research Involving Humans (Guo Wei Ke Jiao Fa 2023 No. 4) [in Chinese]. Central People's Government of the People's Republic of China.

[43] Yang S, Bui CN, Park K (2023). Mobile health apps for breast cancer: content analysis and quality assessment. JMIR mHealth uHealth.

[44] Chasiotis G, Stoyanov SR, Karatzas A, Gravas S (2023). Greek validation of the user version of the Mobile Application Rating Scale (uMARS). J Int Med Res.

[45] Domnich A, Arata L, Amicizia D, Signori A, Patrick B, Stoyanov S, Hides L, Gasparini R, Panatto D (2016). Development and validation of the Italian version of the Mobile Application Rating Scale and its generalisability to apps targeting primary prevention. BMC Med Inf Decis Making.

[46] Hee Ko KK, Kim SK, Lee Y, Lee JY, Stoyanov SR (2022). Validation of a Korean version of Mobile App Rating Scale (MARS) for apps targeting disease management. Health Informatics J.

[47] Messner EM, Terhorst Y, Barke A, Baumeister H, Stoyanov S, Hides L, Kavanagh D, Pryss R, Sander L, Probst T (2020). The German version of the Mobile App Rating Scale (MARS-G): development and validation study. JMIR mHealth uHealth.

[48] Miró J, Llorens-Vernet P (2021). Assessing the quality of mobile health-related apps: interrater reliability study of two guides. JMIR mHealth uHealth.

[49] Shen C, Jiang B, Yang Q, Wang C, Lu KZ, Gu M, Yuan J (2021). Mobile apps for drug-drug interaction checks in Chinese app stores: systematic review and content analysis. JMIR mHealth uHealth.

[50] Schmeelk S, Davis A, Li Q, Shippey C, Utah M, Myers A, Reading Turchioe M, Masterson Creber R (2022). Monitoring symptoms of COVID-19: review of mobile apps. JMIR mHealth uHealth.

[51] Mani M, Kavanagh DJ, Hides L, Stoyanov SR (2015). Review and evaluation of mindfulness-based iPhone apps. JMIR mHealth uHealth.

[52] Amor-García MÁ, Collado-Borrell R, Escudero-Vilaplana V, Melgarejo-Ortuño A, Herranz-Alonso A, Arranz Arija JÁ, Sanjurjo-Sáez M (2020). Assessing apps for patients with genitourinary tumors using the Mobile Application Rating Scale (MARS): systematic search in app stores and content analysis. JMIR mHealth uHealth.

[53] Yamamoto K, Ito M, Sakata M, Koizumi S, Hashisako M, Sato M, Stoyanov SR, Furukawa TA (2022). Japanese version of the Mobile App Rating Scale (MARS): development and validation. JMIR mHealth uHealth.

[54] Bricca A, Pellegrini A, Zangger G, Ahler J, Jäger M, Skou ST (2022). The quality of health apps and their potential to promote behavior change in patients with a chronic condition or multimorbidity: systematic search in app store and Google Play. JMIR mHealth uHealth.

[55] Kim BY, Sharafoddini A, Tran N, Wen EY, Lee J (2018). Consumer mobile apps for potential drug-drug interaction check: systematic review and content analysis using the Mobile App Rating Scale (MARS). JMIR mHealth uHealth.

[56] Santo K, Richtering SS, Chalmers J, Thiagalingam A, Chow CK, Redfern J (2016). Mobile phone apps to improve medication adherence: a systematic stepwise process to identify high-quality apps. JMIR mHealth uHealth.

[57] Romero RL, Kates F, Hart M, Ojeda A, Meirom I, Hardy S (2019). Quality of deaf and hard-of-hearing mobile apps: evaluation using the Mobile App Rating Scale (MARS) with additional criteria from a content expert. JMIR mHealth uHealth.

[58] Narrillos-Moraza Á, Gómez-Martínez-Sagrera P, Amor-García MÁ, Escudero-Vilaplana V, Collado-Borrell R, Villanueva-Bueno C, Gómez-Centurión I, Herranz-Alonso A, Sanjurjo-Sáez M (2022). Mobile apps for hematological conditions: review and content analysis using the Mobile App Rating Scale. JMIR mHealth uHealth.

[59] Báez GN, Rodríguez RH, Fernández GM, Abdel-Kader ML (2022). Smartphone apps for patients with hematologic malignancies: systematic review and evaluation of content. JMIR mHealth uHealth.

[60] Liu X, Jin F, Hsu J, Li D, Chen W (2021). Comparing smartphone apps for traditional Chinese medicine and modern medicine in China: systematic search and content analysis. JMIR mHealth uHealth.

[61] Cheah KJ, Manaf ZA, Ludin AFM, Razalli NH, Mohd Mokhtar N, Md Ali SH (2024). Mobile apps for common noncommunicable disease management: systematic search in app stores and evaluation using the Mobile App Rating Scale. JMIR mHealth uHealth.

[62] Bardus M, Awada N, Ghandour LA, Fares EJ, Gherbal T, Al-Zanati T, Stoyanov SR (2020). The Arabic version of the Mobile App Rating Scale: development and validation study. JMIR mHealth uHealth.

[63] Bardus M, van Beurden SB, Smith JR, Abraham C (2016). A review and content analysis of engagement, functionality, aesthetics, information quality, and change techniques in the most popular commercial apps for weight management. Int J Behav Nutr Phys Act.

[64] Greckhamer T, Furnari S, Fiss PC, Aguilera RV (2018). Studying configurations with qualitative comparative analysis: best practices in strategy and organization research. Strategic Organ.

[65] Greckhamer T, Misangyi VF, Fiss PC (2013). The two QCAs: from a small-N to a large-N set theoretic approach. Res Sociol Organ.

[66] Ragin CC (2008). Redesigning Social Inquiry: Fuzzy Sets and Beyond.

[67] Schneider CQ, Wagemann C (2012). Set-Theoretic Methods for the Social Sciences: A Guide to Qualitative Comparative Analysis.

[68] Benjumea J, Ropero J, Rivera-Romero O, Dorronzoro-Zubiete E, Carrasco A (2020). Assessment of the fairness of privacy policies of mobile health apps: scale development and evaluation in cancer apps. JMIR mHealth uHealth.

[69] Benjumea J, Ropero J, Rivera-Romero O, Dorronzoro-Zubiete E, Carrasco A (2020). Privacy assessment in mobile health apps: scoping review. JMIR mHealth uHealth.

[70] Papageorgiou A, Strigkos M, Politou E, Alepis E, Solanas A, Patsakis C (2018). Security and privacy analysis of mobile health applications: the alarming state of practice. IEEE Access.

[71] Huckvale K, Torous J, Larsen ME (2019). Assessment of the data sharing and privacy practices of smartphone apps for depression and smoking cessation. JAMA Netw Open.

[72] Fiss PC (2007). A set-theoretic approach to organizational configurations. Acad Manage Rev.

[73] Ragin CC (2006). Set relations in social research: evaluating their consistency and coverage. Polit Anal.

[74] Pappas IO, Woodside AG (2021). Fuzzy-set qualitative comparative analysis (fsQCA): guidelines for research practice in information systems and marketing. Int J Inf Manage.

[75] Dul J (2016). Necessary condition analysis (NCA): logic and methodology of "necessary but not sufficient" causality. Organ Res Methods.

[76] Fiss PC (2011). Building better causal theories: a fuzzy set approach to typologies in organization research. AMJ.

[77] Schneider CQ, Wagemann C (2010). Standards of good practice in qualitative comparative analysis (QCA) and fuzzy-sets. Comp Sociol.

[78] Bland JM, Altman DG (1986). Statistical methods for assessing agreement between two methods of clinical measurement. Lancet.

[79] Schnall R, Rojas M, Bakken S, Brown W, Carballo-Dieguez A, Carry M, Gelaude D, Mosley JP, Travers J (2016). A user-centered model for designing consumer mobile health (mHealth) applications (apps). J Biomed Inf.

[80] Powell AC, Torous J, Chan S, Raynor GS, Shwarts E, Shanahan M, Landman AB (2016). Interrater reliability of mHealth app rating measures: analysis of top depression and smoking cessation apps. JMIR mHealth uHealth.

[81] Mohr DC, Schueller SM, Montague E, Burns MN, Rashidi P (2014). The behavioral intervention technology model: an integrated conceptual and technological framework for eHealth and mHealth interventions. J Med Internet Res.

[82] Torous J, Nicholas J, Larsen ME, Firth J, Christensen H (2018). Clinical review of user engagement with mental health smartphone apps: evidence, theory and improvements. Evid Based Ment Health.

[83] Martínez-Pérez B, de la Torre-Díez I, López-Coronado M (2015). Privacy and security in mobile health apps: a review and recommendations. J Med Syst.

